# Genetic Analysis of Lodging Resistance in 1892S Based on the T2T Genome: Providing a Genetic Approach for the Improvement of Two-Line Hybrid Rice Varieties

**DOI:** 10.3390/plants14121873

**Published:** 2025-06-18

**Authors:** Wei Zhang, Liang Zhou, Dahu Ni, Jinlong Ni, Fengshun Song, Liansong Yang, Dewen Zhang

**Affiliations:** Anhui Province Key Laboratory of Rice Genetics and Breeding, Rice Research Institute, Anhui Academy of Agricultural Sciences, Hefei 230031, China; zhangw@aaas.org.cn (W.Z.);

**Keywords:** telomere-to-telomere genome assembly, two-line sterile line (1892S), lodging resistance genes, comparative genomics, hybrid rice breeding

## Abstract

Successfully breeding high-yield, lodging-resistant hybrid rice varieties is critical for ensuring food security. Two-line hybrid rice system plays an essential role in rice breeding, and 1892S, an important two-line sterile line, has contributed significantly to the development of over 100 hybrid rice varieties with superior agronomic traits, including lodging resistance. Despite its importance, a comprehensive understanding of the genomic basis underlying these traits in 1892S has been lacking due to the limitations of short-read sequencing technologies. To address this gap, we utilized advanced telomere-to-telomere (T2T) genome assembly techniques to generate a high-quality, gap-free genome of 1892S—the final genome comprises 12 complete chromosomes with 40,560 protein-coding genes. Comparative genomic analysis identified multiple known lodging resistance genes, including *SD1*, *Sdt97*, *SBI*, *OsFBA2*, *APO1*, and *OsTB1*, with unique allelic variations that may enhance resistance. The pan-genome analysis identified 2347 strain-specific genes in 1892S, further supporting its unique genetic advantages. This study represents the complete T2T genome assembly of a two-line sterile line and provides novel insights into the genetic foundation of lodging resistance in hybrid rice. This study highlights the genetic potential of 1892S in hybrid rice breeding and provides a model for the genomic analysis of other two-line sterile lines, offering valuable insights for improving in hybrid rice, including traits lodging resistance, yield stability, and adaptability, which are crucial for global food security.

## 1. Introduction

As one of the world’s most important staple crops, rice is pivotal in ensuring global food security [1]. Enhancing both the yield and quality of rice remains a strategic priority for safeguarding the global food supply, particularly in the face of rising population pressures and escalating environmental challenges [2]. Hybrid rice technology has significantly advanced rice productivity, with the two-line male sterile system standing out as a critical innovation [3,4]. These systems not only simplify the hybridization process but also amplify hybrid vigor and yield potential [5].

The advent of genomic tools and resources has further revolutionized hybrid rice breeding. Recognizing the importance of unraveling the genomic traits of hybrid rice parents, including two-line male sterile lines, researchers have increasingly adopted comprehensive genomic and phenotypic analyses at the whole-genome level [6,7]. Such analyses provide a theoretical framework for precisely breeding hybrid rice varieties. Quantitative trait nucleotide (QTN) loci, which often reside in highly conserved regions and overlap with open chromatin domains, hold promise for identifying functional sites of candidate genes [8]. Innovations like *RiceNavi*, a genomic navigation system, leverage next-generation sequencing (NGS) data to analyze QTN loci, evaluate breeding strategy success rates, and identify optimal genotypes [9]. Further complementing these advances, third-generation sequencing (TGS) technologies, such as PacBio HiFi and Oxford Nanopore Technologies (ONT), facilitate the assembly of complete genomes with unmatched accuracy, significantly propelling rice genetic breeding [10].

These technological breakthroughs have enabled deeper insights into the genomic foundations of hybrid rice breeding. For instance, Zhang et al. assembled the genomes of multiple two-line and three-line sterile lines, revealing that sterile lines exhibit fewer structural variants (SVs) than restorer lines, with two-line sterile lines displaying the least SVs [11]. This finding underscores the genetic stability of two-line sterile lines and their utility in hybrid breeding. Nevertheless, despite their critical role, comprehensive genomic analyses of two-line sterile lines at the whole-genome level remain sparse, presenting a significant gap in our understanding of their genetic and functional traits.

A notable representative of two-line sterile lines is 1892S, which has profoundly influenced hybrid rice development. As the maternal line for over 100 hybrid rice varieties, 1892S has demonstrated desirable traits such as high yield, strong tillering ability, and excellent lodging resistance [12]. Additionally, many other sterile lines have been developed by modifying 1892S, emphasizing its foundational role in rice breeding. While high-throughput sequencing technologies have been employed to identify beneficial alleles in 1892S, their inherent limitations, including short read lengths and an inability to resolve complex genomic regions, have hindered efforts to fully elucidate its genetic makeup.

To address these challenges, this study employed telomere-to-telomere (T2T) genome assembly technology, integrating long-read sequencing data from PacBio HiFi and ONT platforms with Hi-C chromosomal conformation data [13]. This approach enabled the construction of a high-quality, gap-free genome for 1892S, offering unprecedented genomic resolution. The comprehensive genome was further annotated for protein-coding genes, followed by comparative analyses and detailed investigations of genes associated with lodging resistance.

Lodging poses a significant constraint in rice cultivation, adversely affecting grain yield, quality, and harvesting efficiency. Understanding the genetic basis of lodging resistance is thus imperative for breeding robust rice varieties. This study identified multiple known lodging resistance genes in the 1892S genome, including *SD1*, *Sdt97*, *SBI*, *OsFBA2*, *APO1*, and *OsTB1*, along with unique allelic variations that may confer enhanced resistance [14,15,16,17,18,19]. These findings provide critical insights into the molecular mechanisms underpinning lodging resistance in rice.

In summary, this study presents a comprehensive T2T genome assembly of a two-line sterile line, delivering a high-precision genomic blueprint of 1892S. The results not only enrich the genetic resources available for hybrid rice improvement but also establish a robust platform for future functional genomic studies. By elucidating the genetic architecture of 1892S, this research paves the way for the development of high-yielding, stress-tolerant hybrid rice varieties, contributing to sustainable agriculture and global food security.

## 2. Results

### 2.1. A Telomere-to-Telomere Gap-Free Genome for 1892S

**Figure 1:**
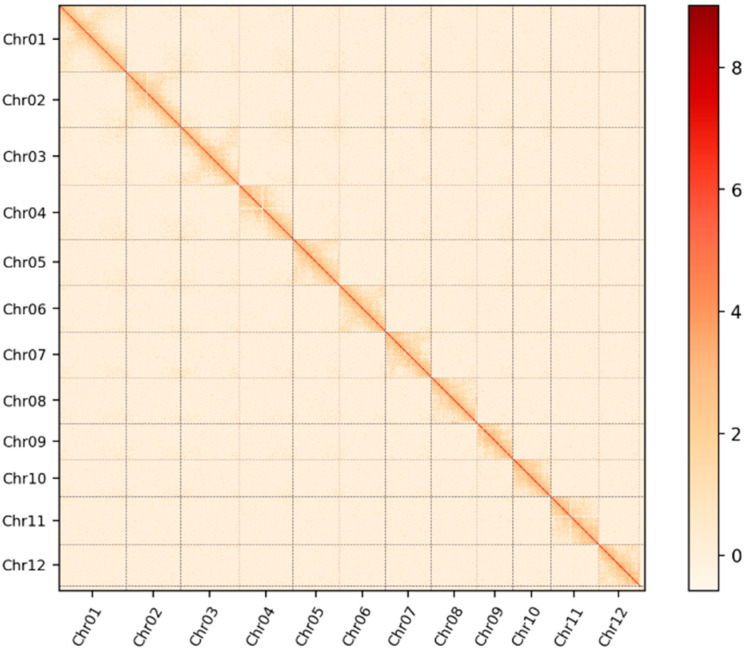
Hi-C interaction heatmap of the 1892S genome. Both the X and Y axes represent genomic positions divided into 100 kb bins (N × 100 kb), and each axis follows chromosome order from Chr01 to Chr12. Color gradients indicate the frequency of interaction between genomic regions, with darker colors indicating stronger chromatin contacts. Note: Although chromatin interaction frequencies in this map can reach values as high as 8, most visible intensities fall within the 2–3 range due to figure rendering limits. Strongest interactions are observed along the diagonal (i.e., between adjacent regions on the same chromosome), as expected. The lower interaction intensities in off-diagonal regions indicate minimal inter-chromosomal noise, which in turn reflects the high accuracy and contiguity of the T2T genome assembly.

The temperature-sensitive two-line sterile line, 1892S, was employed for T2T (telomere-to-telomere) genome assembly. A total of 17.58 Gb (18,876,179,922 bp, ≥10,000 bp, approximately 43.95× coverage) of HiFi reads were generated using the PacBio platform, while 8.08 Gb (8,673,538,823 bp, ≥50,000 bp, approximately 20× coverage) ultra-long ONT reads were produced with the Oxford Nanopore Technology (ONT) platform. In the initial assembly stage, Hifiasm was employed to merge HiFi, ultra-long ONT reads, and Hi-C reads, a strategy that guaranteed the accuracy of the assembled genome. The assembled genome had a total size of 404,410,499 bp, with an N50 size of 31,773,460 bp and a GC content of 43.67%. Hi-C sequencing data was then integrated using the 3D-DNA and Juicer pipelines to facilitate the contigs into chromosomes. After refinement with Juicer-Box, the genome assembly was consolidated into 12 contigs, corresponding to the 12 chromosomes. To ensure a gap-free genome, we used ultra-long ONT reads to close residual gaps. The finalized assembly was compared to the Nipponbare T2T genome using MUMmer, confirming the accuracy of the chromosomes’ number [13] (Figure 1). This comprehensive assembly process successfully generated a high-quality, complete genome for 1892S, serving as a robust foundation for further genetic and functional analyses (Table 1).

**Figure 2:**
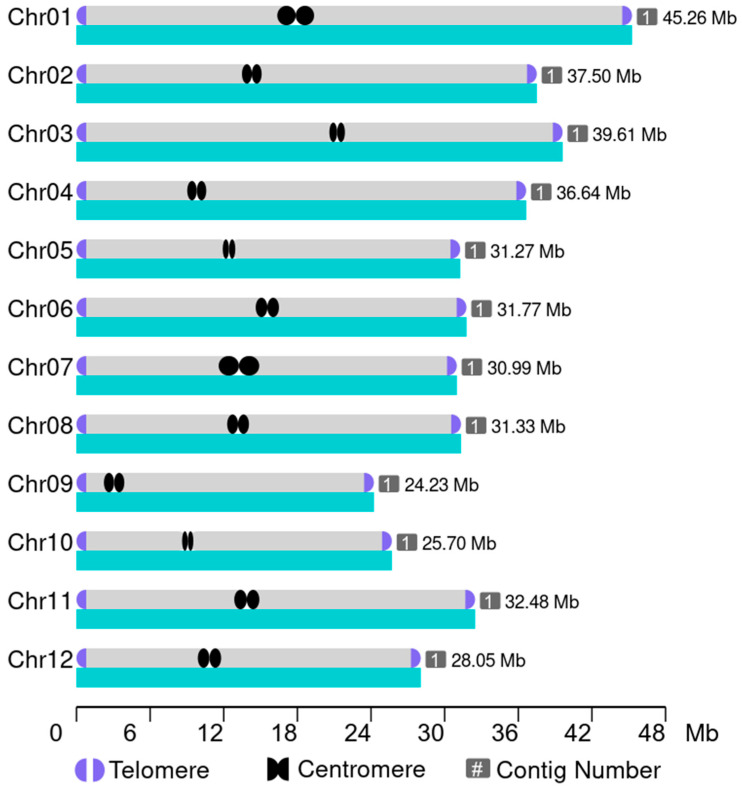
This figure illustrates an overview of the high-contiguity the genome assembly of the 1892S, in which each of the 12 chromosomes is assembled as a single contig without gaps. Telomeric repeat sequences have been identified at both ends of all chromosomes, and centromeric regions have been predicted based on repeat density and sequence characteristics.

Using the characteristic repeat sequences of telomeres (TTTAGGG/TCA), the entire genome was systematically scanned. For each chromosome, regions with at least four repeats of these sequences within the 50 kb flanking both chromosome ends were identified and quantified, as shown in Figure 2. Additionally, HiCAT analysis successfully identified 12 centromeres across the genome. The arrangement of contigs on the chromosomes was visualized, with clear annotations marking the positions of telomeres and centromeres (Figure 2). Ultimately, the complete assembly of the 1892S genome was achieved, comprising 12 chromosomes, 24 telomeres, and 12 centromeres, with a total genome size of 394,819,109 bp (Figure 2, Table 1).

### 2.2. T2T Genome Assembly Evaluation of 1892S

**Figure 3:**
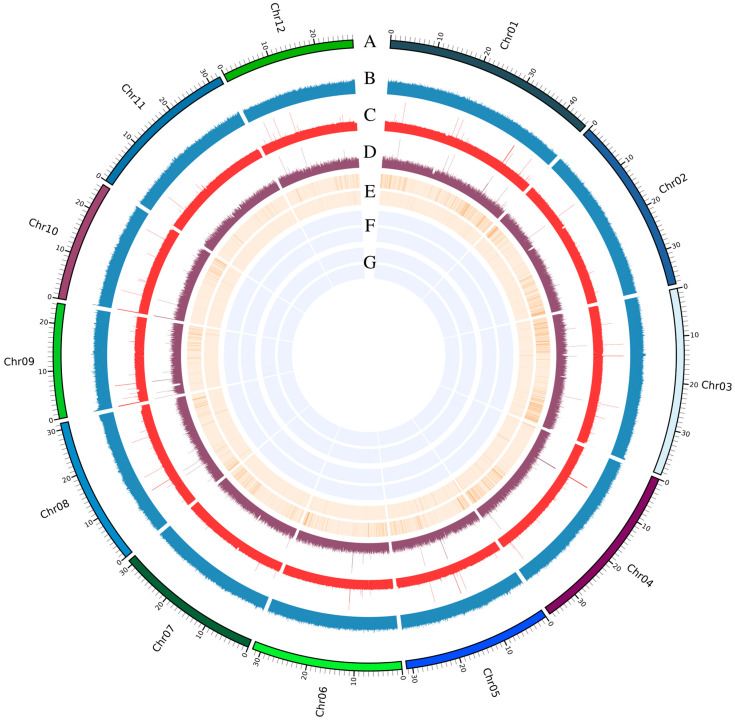
Comprehensive genomic quality assessment of the 1892S genome assembly, including assembly metrics, sequencing depth, and variant distribution profiles. A. Summary of genome assembly statistics, including genome size. B. Distribution of GC content across the genome, indicating regional variation in nucleotide composition. C. Sequencing depth distribution of Illumina short reads mapped to the 1892S genome. D. Sequencing depth distribution of long reads (PacBio HiFi and ultra-ONT) across the genome. E. BUSCO gene completeness assessment: the outer circle shows the distribution of single-copy BUSCOs, while the inner circle represents duplicated BUSCOs, demonstrating gene completeness and copy number consistency. A complete F. SNP density distribution: the outer ring displays homozygous SNP density, and the inner ring shows heterozygous SNP density across the genome. G. InDel density distribution: the outer ring displays homozygous InDel density, and the inner ring shows heterozygous InDel density. These multi-layered metrics collectively support the high quality and accuracy of the 1892S genome assembly.

The genome sequence was divided into 10 kb fragments, and each fragment was aligned to the NCBI nucleotide database (NT library) using BLAST (BLAST 2.15.0+). The analysis revealed that 99.93% of the sequences were identified as belonging to *Oryza*, accounting for 99.98% of all comparisons, with a median alignment consistency of 99.99%. To further evaluate genome quality, short-read data were aligned to the genome using BWA, achieving a comparison rate of 99.21% and a coverage rate of 99.92%. Similarly, long-read data were aligned using Minimap2, resulting in a comparison rate of 99.90% and a coverage rate of 99.99%. BUSCO analysis demonstrated a genome completeness of 99.19%, indicating high integrity and robustness of the assembly. Additionally, short and long reads were mapped to the genome to calculate homozygous and heterozygous rates for SNPs and InDels. Both homozygous and heterozygous SNP rates were 0.000%, while homozygous and heterozygous InDel rates were 0.001%, reflecting exceptional genome accuracy. Furthermore, the QV (quality value) of 46.492, calculated using Merqury based on short-read data, confirmed the high accuracy and reliability of the assembly. A comprehensive summary of the genome evaluation results for 1892S is presented in Figure 3.

### 2.3. Coding Gene Annotation of 1892S Genome

Repetitive sequences in the 1892S genome were initially identified de novo using the RepeatModeler tool. The output from RepeatModeler was subsequently processed by RepeatMasker, which quantified and masked repetitive elements, resulting in an annotated genome sequence with repetitive regions highlighted. The annotation process incorporated RNA-seq data and gene structures from NIP-T2T and R498 genomes. The initial protein-coding gene annotation was generated using BRAKER, with input data consisting of: (i) PASA-trained gene models; (ii) intron hints derived from aligned RNA-seq reads, and de novo assembled transcripts; (iii) protein hints obtained by mapping UniProt, NIP-T2T, and R498 protein sequences; and (iv) repeat-masked genome data. Subsequently, EVM was employed to integrate various evidence types, including BRAKER-predicted gene models, full-length sequences, protein sequences, de novo transcripts, and genome-guided transcripts, into consensus gene models. In the final stage, PASA was used to refine EVM predictions by adding UTR annotations, predicting gene coding regions, and capturing transcript isoforms. This comprehensive annotation pipeline resulted in the identification of 40,560 protein-coding genes and 53,849 mRNAs, including isoforms, in the 1892S genome. To assess the completeness of genome assembly and annotation, BUSCO was employed, which leverages an evolutionarily conserved gene set as a benchmark. A BUSCO score of 94.00% (of 1614 genes) was achieved, indicating a high level of completeness in genome annotation, signifying the reliability of the predicted gene models.

**Figure 4:**
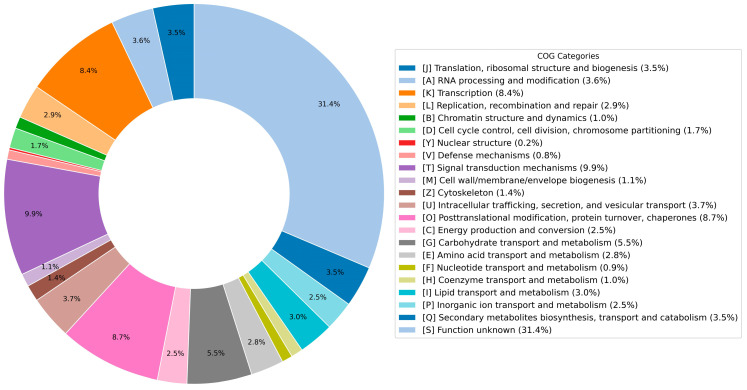
Functional Annotation of the 1892S Genome Based on Clusters of Orthologous Groups (COG) Classification. The bar chart summarizes the distribution of predicted protein-coding genes in 1892S across standardized COG categories. Each bar represents a functional class, annotated using single-letter COG codes. The COG classification provides insight into the functional composition of the genome and the major biological processes represented.

EggNOG-mapper was utilized to allocate COG categories, Gene Ontology (GO) categories, and KEGG pathway annotations to all annotated loci. In the EggNOG database, Orthologous Groups (OGs) serve as a proxy for gene families and are instrumental in functional annotation, classification, and evolutionary studies. Among the sequences analyzed, 41,939 mRNAs were successfully mapped to distinct eggNOG_OGs. The annotated genes were assigned putative functions according to the COG database, and these annotations were subsequently visualized using Python (Python 3.9) scripts (Figure 4).

### 2.4. Detection of 1892S Lodging Resistance Genes

According to the literature, rice lodging resistance-related genes were identified, and further analysis revealed that 1892S harbors six key lodging resistance genes (Table 2). Spontaneous mutations in *SD1* predominantly occur in the first and second exon regions [14]. Sequence alignment showed that the *sd1* genotype in 1892S corresponds to the 93-11 genotype (Supplemental File S1). Additionally, a point mutation was identified in the 5’ untranslated region (UTR) of the rice semi-dwarfing gene *Sdt97*, involving a G-to-C transversion at the 59-UTR site [15]. In 1892S, the *Sdt97* gene exhibits a C at this specific site, confirming a mutation in its *Sdt97* sequence (Supplemental File S2). The amino acid at position 338 in the conserved region of the SBI protein sequence plays a critical role in determining enzyme activity, with arginine (R) at this position [16]. Sequence analysis showed that the *SBI* gene in 1892S aligns perfectly with the corresponding protein sequence in the R498 variety, confirming that 1892S contains a functional *SBI* gene (Supplemental File S3). Moreover, the promoter region of the *OsFBA2* gene, located at position 5,248,026 on chromosome 7 of Nipponbare, contains a T nucleotide. This SNP at position 5,248,026 is functional, and the *OsFBA2* gene is classified as haplotype 3 (hap3) [17]. The corresponding site in 1892S also has a T, indicating that *OsFBA2* in 1892S functions as a lodging resistance gene (Supplemental File S4). For the *scm2* candidate gene *APO1*, a 9 bp deletion in the second exon was identified [18]. The corresponding gene in 1892S similarly exhibits a 9 bp deletion in the same exon (Supplemental File S5). Additionally, compared to the Nipponbare genome, the *scm3* candidate gene *OsTB1* has a 4 bp insertion with the sequence “TGTG” [19]. The corresponding *OsTB1*gene in 1892S also contains an identical 4 bp insertion (Supplemental File S6). These results collectively demonstrate that 1892S carries multiple lodging resistance genes, highlighting its potential as a valuable genetic resource for improving rice lodging resistance.

### 2.5. Lodging Resistance Evaluation and Validation of 1892S-Based Hybrid Rice Varieties

1892S is widely used in practical production, and there are more than 100 hybrid rice varieties nationwide with 1892S as the female parent. The hybrid varieties with 1892S as the female parent also exhibit strong lodging resistance. In this study, using the method of identifying lodging resistance (DB34/T 3924-2021), the lodging resistance of multiple varieties was identified. The results showed that the three varieties with 1892S as the female parent, Wandao 153 (CK), Huiliangyou 27 Zhan, Huiliangyou 985, and Huiliangyou Yuehesimiao, all have strong lodging resistance (Table 3).

### 2.6. Domain-Pan-Genome Dual Screening for Lodging Resistance-Specific Genes

Pan-genome analysis revealed 2,347 strain-specific genes in the 1892S genome, absent in other rice accessions (Supplemental File S7). Pan-genome and whole-genome data integration identified 35 specific genes within lodging resistance-related gene families (Supplemental File S8). Both *SD1* and *SBI* encode proteins containing the DIOX_N and 2OG-FeII_Oxy domains, which are potentially involved in gibberellin biosynthesis regulation (Table 2). Notably, two strain-specific genes in 1892S also harbor these dual domains, suggesting functional conservation or neofunctionalization within this gene family. The *SCM2* gene contains an F-box domain, a structural feature linked to cell wall remodeling through ubiquitin-mediated proteolysis. Strikingly, 32 strain-specific genes in 1892S were identified to possess F-box domains, indicating a significant expansion of this functional module. The *SDT97* gene carries an Adenine_glyco domain, associated with delayed senescence via adenine glycosylation. A single strain-specific gene in 1892S was found to retain this domain, highlighting its potential role in senescence regulation.

## 3. Discussion

This study presents the first telomere-to-telomere (T2T) genome assembly of the two-line hybrid rice sterile line 1892S, providing a high-resolution reference for functional genomic research and trait-associated variant discovery. In two-line hybrid rice breeding systems, male sterility is conferred by photoperiod- or thermo-sensitive genic male sterile (P/TGMS) lines. 1892S is a thermo-sensitive genic male sterile (TGMS) line, which becomes male sterile when grown at temperatures above approximately 23.5 °C. Under such conditions, it serves as the female parent in hybrid seed production. When grown under cooler conditions below this threshold, fertility is restored, enabling self-pollination and seed propagation. Hybrid varieties are developed by crossing TGMS lines with fertile pollen parents.

We successfully achieved a high-precision, gap-free genome assembly by leveraging advanced sequencing technologies, including PacBio HiFi, ultra-long Oxford Nanopore Technology (ONT) reads, and Hi-C data. The assembly obtained an exceptional BUSCO completeness score of 99.19% and a QV value of 46.492, demonstrating the robustness and reliability of the assembled genome. This high-quality reference genome provides a valuable resource for functional genomics research, gene discovery, and precision breeding aimed at enhancing hybrid rice traits [20,21].

To identify superior and detrimental genes at the whole-genome level, we employed the *RiceNavi* system to predict key genetic loci based on second-generation sequencing data. Additionally, second-generation sequencing data from 1892S were analyzed to predict superior alleles [12]. In this study, the 1892S T2T genome served as a reference for genotype analysis based on previously reported genes, revealing more detailed insights compared to previous research. For instance, the identification of lodging resistance genes in 1892S demonstrates the genome’s capacity to provide in-depth analyses of genes associated with this critical trait.

Lodging is a significant agronomic challenge that negatively impacts rice yield, quality, and harvesting efficiency [22]. Our study identified six key lodging resistance genes within the 1892S genome: *SD1*, *Sdt97*, *SBI*, *OsFBA2*, *APO1*, and *OsTB1*, which operate through interconnected molecular pathways, collectively enhancing stem strength and optimizing plant architecture. *SD1* encodes GA 20-oxidase, and its loss-of-function mutation (such as the 93-11 allele present in 1892S) reduces bioactive gibberellin levels, leading to semi-dwarf stature and reduced lodging risk [23]. *Sdt97*, carrying a G-to-C transversion in its 5′ UTR, likely fine-tunes GA biosynthesis post-transcriptionally, providing a balanced regulation of internode elongation and stem stability [24]. Together, these alleles reflect a dual mechanism in GA homeostasis, enhancing plant robustness without significantly compromising yield potential [25]. *SBI* restricts the elongation of basal internodes and enhances cell wall rigidity by promoting GA 2-oxidase activity, which increases lignin deposition and secondary wall thickening [25]. *APO1*, which contains an F-box domain, is thought to regulate the stability of cell wall remodeling proteins. The 9-bp exon deletion identified in *APO1* in 1892S may alter its protein interaction dynamics, potentially stabilizing proteins that strengthen fiber alignment, a feature commonly associated with stem mechanical strength in cereals [26]. *OsTB1* functions as a transcriptional repressor of tiller bud outgrowth. The 4-bp insertion detected in 1892S may enhance its repression activity, resulting in a reduced tiller angle and a more compact canopy that lowers wind-induced mechanical stress [27]. *OsFBA2*, another F-box gene, contains a promoter SNP (hap3) that may influence photoperiod-sensitive tillering through interaction with florigen-related signaling pathways [28]. These genetic modifications contribute to an upright plant type with improved lodging tolerance while maintaining efficient light capture. Taken together, these gene variants act synergistically, targeting hormone signaling, structural reinforcement, and architectural regulation—key components of a multifaceted genetic strategy against lodging in 1892S. Collectively, these functionally validated genes represent key genetic components of a robust lodging resistance network in 1892S, integrating hormonal, structural, and architectural regulatory mechanisms. Compared to previous studies [12], this research introduces several novel contributions. Both *Sdt97* and *OsFBA2* were identified as lodging resistance genes through the analysis of the 1892S genome, a discovery not found in earlier studies. This difference is likely due to the methodological approach: while *RiceNavi* primarily relies on SNP loci, our study leveraged complete gene sequences, including both upstream and downstream regions.

In addition to the analysis of previously reported lodging resistance genes, we also performed a pan-genome-based comparative analysis. Using the core genome defined in a published rice of pan-genome dataset as reference, we predicted 2347 protein-coding genes in 1892S. 35 of them contain conserved domains associated with lodging resistance gene families, suggesting their potential involvement in the regulation of lodging-related traits. These candidates may contribute to the superior performance of 1892S in hybrid combinations. However, further functional characterization is necessary to confirm their biological roles and relevance to lodging resistance.

The presence of multiple lodging resistance genes in 1892S genome might play a significant role in the development of over 100 hybrid rice varieties, manyof which exhibit superior lodging resistance agronomic traits. Field evaluations of hybrid rice varieties derived from 1892S demonstrated outstanding lodging resistance and adaptability to diverse environmental conditions. These results underscore the practical value of 1892S as a maternal line in hybrid rice breeding.

This study provides a comprehensive genomic analysis of 1892S, uncovering the genetic basis of lodging resistance, and offers a model for analyzing other traits, such as nitrogen efficiency and high yield. However, some limitations must be acknowledged. For instance, trait analysis was based on previously reported genes studied in other experimental materials. Genetic differences between materials may exist, implying that 1892S might harbor additional beneficial genes not yet identified. As such, this method may not fully capture all potential advantageous genes in 1892S.

This study not only examines lodging resistance-related genes in 1892S using the T2T genome as a reference but also provides a model for the analysis of other two-line sterile lines and rice varieties. By utilizing chromosome-level parental genomes as references and integrating previously reported functional genes, this approach enables the identification of both advantageous and disadvantageous alleles in parental lines.

Two-line sterile lines play an essential role in hybrid rice breeding, with their unique traits and superior alleles significantly contributing to the improvement of hybrid rice [29]. By analyzing the characteristics of two-line sterile lines, valuable genetic foundations can be established to achieve high yield, superior quality, and enhanced stress resistance in hybrid rice [30]. The discovery and utilization of superior alleles are critical for improving rice yield, disease resistance, and adaptability. Through the genomic analysis of two-line sterile lines, key alleles associated with important agronomic traits can be identified. These alleles can be incorporated into hybrid rice varieties using molecular breeding techniques, thereby improving overall performance. This integrative genomic approach lays the groundwork for molecular breeding strategies targeting lodging resistance and other agronomic traits in hybrid rice.

## 4. Materials and Methods

### 4.1. Genomic DNA and RNA Extraction Sequencing

Harvested with precision, the vibrant fresh leaves of the 1892S cultivar were swiftly immersed in liquid nitrogen to ensure their preservation. Total RNA was extracted from three biological replicates, each consisting of leaves collected from independent three-week-old 1892S seedlings, using the TRIzol™ Reagent (Invitrogen, Carlsbad, CA, USA) following the manufacturer’s protocol, with quality and concentration assessed by spectrophotometry and gel electrophoresis. Utilizing the CTAB extraction protocol, we successfully isolated high-quality genomic DNA from these leaf samples. In pursuit of comprehensive whole genome sequencing (WGS), we crafted a paired-end (300 bp) library using the Nextera DNA Flex Library Prep Kit (Illumina, San Diego, CA, USA) and deployed it for sequencing on the advanced Illumina NovaSeq platform (Illumina, San Diego, CA, USA), thereby securing a dataset of exceptional clarity. For the acquisition of Oxford Nanopore Technologies (ONT) ultra-long reads, we assembled a DNA library with the SQK-LSK110 ligation kit (Nanopore, Oxford, UK). Following meticulous purification, this library was sequenced on the PromethION instrument (Nanopore, Oxford, UK), promising in-depth genomic insights. In our quest for PacBio HiFi sequencing, we constructed a long-read library (15 kb) from the genomic DNA using the SMRTbell^®^ Express Template Prep Kit 2.0 (PacBio, Menlo Park, CA, USA). This library was then processed on a Sequel II instrument (PacBio, USA), delivering high-fidelity sequencing results. For Hi-C sequencing, we prepared a library with the VAHTSTM Fg DNA Library Prep Kit (Novozymes, Nanjing, China) and conducted sequencing on the Illumina NovaSeq X Plus instrument (San Diego, CA, USA), generating 82,983,684 paired-end reads (150 bp). To ensure data integrity, we meticulously filtered out low-quality sequences from the raw data, thereby refining our dataset to its cleanest form.

### 4.2. T2T and Chloroplast Genome Assembly

The contigs were initially assembled using Hifiasm [31] from PacBio HiFi, ONT ultra-long, and Hi-C data, followed by purity identification and de-hybridization with Purge Haplotigswas used to identify and de-hybridy by purge_dups [32]. The Hi-C data were then used for chromosome anchoring and assembly into chromosomes using 3D-DNA and Juicer. The result was manually adjusted using Juicebox. TGS-GapCloser [33] was used to fill the gap between contigs and extend contigs by using the covering relationship between the ONT ultra-long data and the already assembled contigs, followed by comparison with the NIP-T2T genome via Mummer [34] to fix the chromosome number. One polishing was performed based on WGS data by Pilon [35]. Finally, a high-quality and gapless genome was assembled. For chloroplast genome (cpDNA) was assembled by GetOrganelle [36] based on clean WGS data and visualized using Bandage [37]. Annotation was conducted on CPGAVAS2 using the FASTA-formatted circular sequence based on rice (NCBI accession: NC_001320) as the reference genome [38].

### 4.3. Telomere and Centromere Identification

Ultra-long ONT reads larger than 100 kb were compared to chromosomes using Minimap2 [39], and all reads that were compared once within 100 bp of the end of chromosomes were collected. The read containing the artifact sequence is filtered out, and then the comparison of the read is counted, and the read of the extendible median length is defined as a reference and the other is query; medaka_consensu reassembled reference telomere read and query telomere read to obtain a consistent sequence. Blast was used to compare the above consistency sequences to both ends of each chromosome, and the alignment sequences with coverage >= 90 were replaced with the upper-end grain sequences according to the alignment position. Telomere sequences were identified based on their characteristic repeat motifs (CCCTAAA/TAG) using a quartet [40], which efficiently detects these base repeat patterns in genomic regions. To analyze the centromeric regions of the 1892S genome, the HiCAT tool was employed [41], enabling precise centromere identification and characterization.

### 4.4. Genome Assembly Quality Assessment

To validate that the assembly results corresponded to the target species, the genome sequence was fragmented into 10 kb segments and compared against the NCBI nucleotide database (NT library) using BLAST. For alignment, short-read and long-read data were mapped to the genome using BWA and Minimap2, respectively. The comparison rate and coverage rate were calculated, where higher values indicated greater consistency between the assembly and the reads, reflecting better assembly quality. Genome integrity was assessed using BUSCO (Benchmarking Universal Single-Copy Orthologs), which evaluates assembly completeness based on orthologous genes in the OrthoDB database [42]. After aligning short and long reads to the genome, mutations were identified using Samtools [43], Picard [44], and GATK, enabling the calculation of homozygous and heterozygous SNP and InDel rates. Lower homozygous rates indicated higher genome accuracy, while higher heterozygous rates reflected greater genome heterozygosity. Additionally, the assembly accuracy was validated using the QV (quality value) metric provided by Merqury, offering a quantitative assessment of the genome assembly’s precision [45].

### 4.5. Genome Annotation

The genome was soft-masked using RepeatMasker, guided by repetitive elements identified through RepeatModeler [46]. Trinity was employed for the de novo assembly of all RNA-seq reads [47]. These genome-guided assemblies, de novo transcript assemblies, and RNA-seq alignment data were integrated into a comprehensive transcriptome, which served as the foundation for constructing high-confidence gene models using PASA v2.4.1 [48]. Initial gene predictions were generated with BRAKER [49], incorporating multiple layers of evidence: (1) high-confidence gene models from PASA, (2) Intron hints derived from Illumina short-read alignments; (3) Protein hints based on mappings of proteins from NIP-T2T, R498, and UniProt to the genome using Exonerate; (4) The soft-masked genome of 1892S.

EVidenceModeler (EVM) was then utilized to combine gene models from various sources [50], including BRAKER predictions, genome-guided transcripts from Illumina RNA-seq, de novo transcripts, and protein alignments from NIP-T2T, R498, and UniProt. PASA further refined these gene models by annotating alternative splicing events, defining untranslated regions (UTRs), and refining gene structures to produce the final high-quality annotation. Functional annotation of the annotated genes was conducted utilizing the EggNOG-mapper [51].

### 4.6. Gene Retrieval for Lodging Resistance in Rice

Through an extensive search of academic literature on rice lodging resistance, the genes associated with this trait were systematically analyzed. The relevant gene ID and their corresponding sequences were then extracted for further analysis. Translate: Extract the retrieved gene sequences, and map them to the 1892S genome to find the corresponding gene numbers by Minimap2 and BEDTools [52]. Next, further, search the relevant literature, analyze the consistency of the lodging resistance gene sequence alignment and the corresponding genotypes, and then determine whether 1892S has the corresponding lodging resistance gene.

### 4.7. Genome Comparison Analysis

The pan-genome sequences of 111 rice accessions were retrieved as a reference dataset [53]. To classify genes in the 1892S genome, Minimap2 (v2.28, -cx asm20) was employed to align 1892S gene sequences against the core genes of the pan-genome. Genes failing to align with the core gene set were classified as 1892S-specific non-core genes. Protein domains across the 1892S genome were annotated using the Pfam_scan (default parameter) tool based on the Pfam database [54]. Genes harboring structural domains associated with lodging resistance were prioritized as candidate genes. Non-core gene sets from the 1892S pan-genome were integrated with the domain-annotated lodging resistance candidates.

### 4.8. Method for Identifying Rice Lodging Resistance

The lodging resistance of rice varieties was evaluated following the Anhui Provincial Standard DB34/T 3924-2021 [55]. Test and control varieties (selected for strong lodging resistance) were cultivated uniformly under field conditions adhering to NY/T 1300 guidelines. During the dough stage, representative tillers were selected, and border rows were excluded. Custom weight bottles (3.5 cm diameter × 6.5 cm height) filled with iron sand (10–250 g, 10 g intervals) were clamped onto the penultimate node of stems. Lodging incidence was recorded for ≥30 stems per weight level. The Lodging Weight 50 (LW_50_) was calculated using Bliss probit analysis, relating lodging rate to log_10_(weight). The Lodging Index (LI) was derived as the ratio of test-to-control LW_50_, with resistance graded into five levels (LI ≥ 1.20: strong; LI < 0.50: very weak). Statistical validation employed probit regression software, ensuring precision (results to two decimal places).

## 5. Conclusions

This study achieved a high-quality telomere-to-telomere (T2T) genome assembly of the two-line sterile line 1892S, with a total size of 394.82 Mb across 12 gap-free chromosomes. The assembly exhibited exceptional accuracy, supported by a BUSCO completeness score of 99.19% and a quality value (QV) of 46.492. Genome annotation identified 40,560 protein-coding genes, including 2347 strain-specific genes through pan-genome analysis, highlighting 1892S’s unique genetic architecture.

Critical lodging resistance genes, including *SD1*, *Sdt97*, *SB1*, *OsFBA2*, *APO1*, and *OsTB1*, were identified with functional allelic variations. These findings provide a genomic resource for leveraging 1892S in hybrid rice breeding, enabling targeted improvement of lodging resistance. The integration of T2T assembly, functional gene annotation, and pan-genomic insights establishes a model for analyzing other sterile lines, advancing precision breeding strategies to contribute to food security.

## Figures and Tables

**Table 1:** Chromosome Assembly Statistics and Structural Features of the 1892S Genome.

Chromosomes	Length (bp)	Number of Contigs	Number of Gaps	Centromere Location	Telomere Start Repeat Unit Number	Telomere End Repeat Unit Number
Chr01	45,262,897	1	0	16,361,678–19,363,169	1404	1947
Chr02	37,503,140	1	0	13,486,004–15,088,336	1786	2031
Chr03	39,610,054	1	0	20,608,780–21,870,485	1607	195
Chr04	36,637,658	1	0	9,025,928–10,575,559	1995	1121
Chr05	31,267,256	1	0	11,918,420–12,941,910	1808	804
Chr06	31,773,107	1	0	14,604,503–16,500,181	904	1045
Chr07	30,986,196	1	0	11,585,584–14,893,168	1042	556
Chr08	31,327,512	1	0	12,278,862–14,055,598	1392	1655
Chr09	24,231,554	1	0	2,235,737–3,897,225	1367	877
Chr10	25,695,394	1	0	8,608,241–9,522,477	1150	1550
Chr11	32,477,472	1	0	12,858,031–14,900,657	1270	525
Chr12	28,046,869	1	0	9,886,763–11,786,338	815	1302
Pt	134,488	1	0	-	-	-

**Table 2:** Lodging Resistance Gene Identification and Protein Family Analysis in Nipponbare and 1892S.

Gene Name	Gene ID of Nipponbare	Gene ID of 1892S	Reference	Pfams
*sd1*	Os01g0883800	Os1892S01G025560	[14]	DIOX_N, 2OG-FeII_Oxy
*Sdt97*	Os06g0649800	Os1892S06G004570	[15]	Adenine_glyco
*SBI*	LOC_Os05g43880	Os1892S05G000700	[16]	DIOX_N, 2OG-FeII_Oxy
*OsFBA2*	LOC_Os07g09870	Os1892S07G004930	[17]	F-box-like, FBA_1
*APO1*	Os06g0665400	Os1892S06G022880	[18]	F-box
*OsTB1*	Os03g0706500	Os1892S03G036300	[19]	TCP

**Table 3:** Quantitative and Phenotypic Assessment of Lodging Resistance in Crop Varieties: Multi-Replicate Trials and Classification.

Variety Name	I	II	III	Average Value	LI (Lodging Index)	Level	Phenotype
Huiliangyou 27 Zhan	44.8	43.3	51.5	46.5	0.9	2	Moderately strong
21SBC3	25.6	39.3	38.4	34.4	0.7	4	Very weak
21SBC4	59.4	55.3	51.2	55.3	1.1	2	Moderately strong
21SBC5	55.8	52.6	58.4	55.6	1.1	2	Moderately strong
21SBC6	39	41.4	32.3	37.6	0.7	3	Normal
21SBC7	27	28.7	34.4	30.0	0.6	4	Very weak
21SBC8	25.5	30.1	22.1	25.9	0.5	4	Very weak
21SBC9	24.4	23.6	21.2	23.1	0.5	5	Weak
21SBC10	38.8	28.8	35.9	34.5	0.7	4	Very weak
21SBC11	37.2	31.3	33.5	34.0	0.7	4	Very weak
21SBC12	26.1	36.4	35	32.5	0.6	4	Very weak
Huiliangyou 985	43.8	57.6	48.4	49.9	0.99	2	Moderately strong
Wandao 153 (CK)	49.7	49.3	51.6	50.2	1.0	2	Moderately strong
Huiliangyou Yuehesimiao	51.2	58.4	54.2	54.6	1.7	2	Moderately strong

## Data Availability

All datasets have been deposited in the National Genomics Data Center (NGDC) with the following accession codes: the raw data, genome assembly, and gene annotation of ‘1892S’, PRJCA037566.

## Supplementary Materials

The following supporting information can be downloaded at: https://www.mdpi.com/article/10.3390/plants14121873/s1, Supplemental File S1: Sequence alignment of *sd1* between 1892S and 93-11; Supplemental File S2: mutation of *Sdt97* in 1892S; Supplemental File S3: Sequence alignment of *SBI* between 1892S and R498; Supplemental File S4: Sequence characteristics of the *OsFBA2* gene in 1892S; Supplemental File S5: Sequence characteristics with 9 bp deletion of the *scm2* gene in 1892S; Supplemental File S6: Sequence characteristics with 4 bp insertion of the *OsTB1* gene in 1892S; Supplemental File S7: Dispensable genes’ ID in the 1892S genome; Supplemental File S8: 35 specific genes’ ID within lodging resistance-related gene families.
