# Supplementary material for: Genetic Analysis of Lodging Resistance in 1892S Based on the T2T Genome: Providing a Genetic Approach for the Improvement of Two-Line Hybrid Rice Varieties
Source: Plants (Basel). 2025 Jun 18;14(12):1873. doi: 10.3390/plants14121873 (PMC12197197; doi:10.3390/plants14121873)
Supplement: Supplementary file 1 [file plants-14-01873-s001.zip › Supplemental File S1.pdf]

CLUSTALW Result

```
          WARNING: possibly wrong combination

             --------------------------------
             Selected type :     PROTEIN
             Query sequence:     DNA
             --------------------------------
```

[clustalw.aln][clustalw.dnd][readme]

Select tree menu | Exec

```
 CLUSTAL 2.1 Multiple Sequence Alignments


Sequence type explicitly set to Protein
Sequence format is Pearson
Sequence 1: Os1892S01G025560  3273 aa
Sequence 2: 9311              5131 aa
Start of Pairwise alignments
Aligning...



(Partial alignment)
Sequences (1:2) Aligned. Score: 91.3841
Guide tree file created:   [clustalw.dnd]

There are 1 groups
Start of Multiple Alignment

Aligning...
Group 1: Sequences:   2      Score:57135
Alignment Score 24657

CLUSTAL-Alignment file created  [clustalw.aln]
```

---

clustalw.aln

```
CLUSTAL 2.1 multiple sequence alignment


Os1892S01G025560      ------------------------------------------------------------
9311                  AGTGCAAGTAGGGTAAGTCTTGTTTACATCATATCGGTTTCATTTTGGTACACGAATGGA


Os1892S01G025560      ------------------------------------------------------------
9311                  GAAGAAATGAAAGAGATCGAAAAAAGGAAGAGCTCGCGGTGTATCTGTCTCGTAACAGCC


Os1892S01G025560      ------------------------------------------------------------
9311                  CCGGTGTTACACGTGCTCTAAGAGAGATTAATTAAATCGATAAGCTACCAGAGGTTTAGT


Os1892S01G025560      ------------------------------------------------------------
9311                  TTTCCACGTGTTAATTAGATTGGAAAGCGAGAGAAATTAAAAATAGCGAGTAAAAATAGA


Os1892S01G025560      ------------------------------------------------------------
9311                  GATAACCTTATTGCTATTTTGTTTTTTTCCAGCAACAAACTTATCTTTCTGGCTAGTTTA


Os1892S01G025560      ------------------------------------------------------------
9311                  GGCGATCGCTTAGATTCCGCATCGTCCTTTTCACTATTTTTTTTCTGTCAGTGACAATGT
```

```
Os1892S01G025560    ------------------------------------------------------------
9311                GAAAATTTATTGGACAGACGACTAGCTTGTGGTACTAGTTAGGAAATTCCCTAACCTCGA

Os1892S01G025560    ------------------------------------------------------------
9311                TATGAACAACTTACTCAACTCAGTAGAGTAGCAAATGCCCAAGAAAGCCCGAGTCAATCT

Os1892S01G025560    ------------------------------------------------------------
9311                ATTTGGAAATCCAATCTATTTTCTCGTATTCGTGTGGGAAATCAAGCTATACTAGTTGAA

Os1892S01G025560    ------------------------------------------------------------
9311                ATTCACCTAAAGAAATGCACGCACTTCAATATACCAAAATTGCAAAGGAGAATCATTCGA

Os1892S01G025560    ------------------------------------------------------------
9311                TTAATAGTGGAATTCAACCAAGAAATGAAAAGGTATATATAGGAAATGCACTCCAACCAC

Os1892S01G025560    ------------------------------------------------------------
9311                CAACCAATAAGTGATTCCGGGCAATCAATTCTATCCGCGAGTTGTGGGTCTGTTCAGATT

Os1892S01G025560    ------------------------------------------------------------
9311                CATTGTATTAGAACGCGTCACGTAATGGATAGAGTATTATACAACACCATTGGTTTTGCC

Os1892S01G025560    ------------------------------------------------------------
9311                ACTAGTGTTAACTCTAATACATGGGGGCTAGTTTTACCTTTAAACTTGGTCTAAAAGGAT

Os1892S01G025560    ------------------------------------------------------------
9311                GGACATATGGCAATGCAATTGCATGGGGGTCATTGATTCGACCATCATGTCTGTCCAGTG

Os1892S01G025560    ------------------------------------------------------------
9311                GCAACCCCCTCCCTCATCCCCTGTGGTGGGCCCCCCACGGCGCTCGTCTTCTCCCCTGTT

Os1892S01G025560    ---------------------------------------ACACACACACACACACTCACA
9311                ACAAATACCCCACCCTCCTGCCCAGACAGCTCGGCCTGCACACACACACACACACTCACA
                                                           *********************

Os1892S01G025560    CTCACACACGCTCTCAACTCACTCCCGCTCAACACAGCGCTCACTTCTCATCTCCAATCT
9311                CTCACACACGCTCTCAACTCACTCCCGCTCAACACAGCGCTCACTTCTCATCTCCAATCT
                    ************************************************************

Os1892S01G025560    CATGGTGGCCGAGCACCCCACGCCACCACAGCCGCACCAACCACCGCCCATGGACTCCAC
9311                CATGGTGGCCGAGCACCCCACGCCACCACAGCCGCACCAACCACCGCCCATGGACTCCAC
                    ************************************************************

Os1892S01G025560    CGCCGGCTCTGGCATTGCCGCCCCGGCGGCGGCGGCGGTGTGCGACCTGAGGATGGAGCC
9311                CGCCGGCTCTGGCATTGCCGCCCCGGCGGCGGCGGCGGTGTGCGACCTGAGGATGGAGCC
                    ************************************************************

Os1892S01G025560    CAAGATCCCGGAGCCATTCGTGTGGCCGAACGGCGACGCGAGGCCGGCGTCGGCGGCGGA
9311                CAAGATCCCGGAGCCATTCGTGTGGCCGAACGGCGACGCGAGGCCGGCGTCGGCGGCGGA
                    ************************************************************

Os1892S01G025560    GCTGGACATGCCCGTGGTCGACGTGGGCGTGCTCCGCGACGGCGACGCCGAGGGGCTGCG
9311                GCTGGACATGCCCGTGGTCGACGTGGGCGTGCTCCGCGACGGCGACGCCGAGGGGCTGCG
                    ************************************************************

Os1892S01G025560    CCGCGCCGCGGCGCAGGTGGCCGCCGCGTGCGCCACGCACGGGTTCTTCCAGGTGTCCGG
9311                CCGCGCCGCGGCGCAGGTGGCCGCCGCGTGCGCCACGCACGGGTTCTTCCAGGTGTCCGG
                    ************************************************************

Os1892S01G025560    GCACGGCGTCGACGCCGCTCTGGCGCGCGCCGCGCTCGACGGCGCCAGCGACTTCTTCCG
9311                GCACGGCGTCGACGCCGCTCTGGCGCGCGCCGCGCTCGACGGCGCCAGCGACTTCTTCCG
                    ************************************************************

Os1892S01G025560    CCTCCCGCTCGCCGAGAAGCGCCGCGCGCGCCGCGTCCCGGGCACCGTGTCCGGCTACAC
```

```
9311                CCTCCCGCTCGCCGAGAAGCGCCGCGCGCCGCGTCCCGGGCACCGTGTCCGGCTACAC
                    ************************************************************

Os1892S01G025560    CAGCGCCCACGCCGACCGCTTCGCCTCCAAGCTCCCATGGAAGGAGACCCTCTCCTTCGG
9311                CAGCGCCCACGCCGACCGCTTCGCCTCCAAGCTCCCATGGAAGGAGACCCTCTCCTTCGG
                    ************************************************************

Os1892S01G025560    CTTCCACGACCGCGCCGCCGCCCCCGTCGTCGCCGACTACTTCTCCAGCACCCTCGGCCC
9311                CTTCCACGACCGCGCCGCCGCCCCCGTCGTCGCCGACTACTTCTCCAGCACCCTCGGCCC
                    ************************************************************

Os1892S01G025560    CGACTTCGCGCCAATGGGGTAATTAAAACGATGGTGGACGACATTGCATTTCAAATTCAA
9311                CGACTTCGCGCCAATGGGGTAATTAAAACGATGGTGGACGACATTGCATTTCAAATTCAA
                    ************************************************************

Os1892S01G025560    AACAAATTCAAAACACACCGACCGAGATTATGCTGAATTCAAACGCGTTTGTGCGCGCAG
9311                AACAAATTCAAAACACACCGACCGAGATTATGCTGAATTCAAACGCGTTTGTGCGCGCAG
                    ************************************************************

Os1892S01G025560    GAGGGTGTACCAGAAGTACTGCGAGGAGATGAAGGAGCTGTCGCTGACGATCATGGAACT
9311                GAGGGTGTACCAGAAGTACTGCGAGGAGATGAAGGAGCTGTCGCTGACGATCATGGAACT
                    ************************************************************

Os1892S01G025560    CCTGGAGCTGAGCCTGGGCGTGGAGCGAGGCTACTATAGGGAGTTCTTCGCGGACAGCAG
9311                CCTGGAGCTGAGCCTGGGCGTGGAGCGAGGCTACTATAGGGAGTTCTTCGCGGACAGCAG
                    ************************************************************

Os1892S01G025560    CTCAATCATGCGGTGCAACTACTACCCGCCATGCCCGGAGCCGGAGCGGACGCTCGGCAC
9311                CTCAATCATGCGGTGCAACTACTACCCGCCATGCCCGGAGCCGGAGCGGACGCTCGGCAC
                    ************************************************************

Os1892S01G025560    GGGCCCGCACTGCGACCCCACCGCCCTCACCATCCTCCTCCAGGACGACGTCGGCGGCCT
9311                GGGCCCGCACTGCGACCCCACCGCCCTCACCATCCTCCTCCAGGACGACGTCGGCGGCCT
                    ************************************************************

Os1892S01G025560    CGAGGTCCTCGTCGACGGCGAATGGCGCCCCGTCAGCCCCGTCCCCGGCGCCATGGTCAT
9311                CGAGGTCCTCGTCGACGGCGAATGGCGCCCCGTCAGCCCCGTCCCCGGCGCCATGGTCAT
                    ************************************************************

Os1892S01G025560    CAACATCGGCGACACCTTCATGGTAAACCATCTCCTATTCTCCTCTCCTCTGTTCTCCTC
9311                CAACATCGGCGACACCTTCATGGTAAACCATCTCCTATTCTCCTCTCCTCTGTTCTCCTC
                    ************************************************************

Os1892S01G025560    TGCTTCGAAGCAACAGAACAAGTAATTCAAGCTTTTTTTTCTCTCGCGCGCGAAATTGAC
9311                TGCTTCGAAGCAACAGAACAAGTAATTCAAGCTTTTTTTTCTCTCGCGCGCGAAATTGAC
                    ************************************************************

Os1892S01G025560    GAGAAAAATAAGATCGTGGTAGGGGCGGGGCTTTCAGCTGAAAGCGGGAAGAAACCGACC
9311                GAGAAAAATAAGATCGTGGTAGGGGCGGGGCTTTCAGCTGAAAGCGGGAAGAAACCGACC
                    ************************************************************

Os1892S01G025560    TGACGTGATTTCTCTGTTCCAATCACAAACAATGGAATGCCCCACTCCTCCATGTGTTAT
9311                TGACGTGATTTCTCTGTTCCAATCACAAACAATGGAATGCCCCACTCCTCCATGTGTTAT
                    ************************************************************

Os1892S01G025560    GATTTATCTCACATCTTATAGTTAATAGGAGTAAGTAACAAGCTATTGATTTTTTTTTGT
9311                GATTTATCTCACATCTTATAGTTAATAGGAGTAAGTAACAAGCTATTGATTTTTTTTTGT
                    ************************************************************

Os1892S01G025560    TAAAGTTTTTTTAGTTTATCCAAATTTATTGAAAAACTTAGCAACGTTTATAATACCAAA
9311                TAAAGTTTTTTTAGTTTATCCAAATTTATTGAAAAACTTAGCAACGTTTATAATACCAAA
                    ************************************************************

Os1892S01G025560    TTAGTCTCATTTAGTTTAATATTGTATATATTTTGATAATATATTTATGTTATATTAAAA
9311                TTAGTCTCATTTAGTTTAATATTGTATATATTTTGATAATATATTTATGTTATATTAAAA
                    ************************************************************

Os1892S01G025560    ATATTACTATATTTTACTATAAACATTATTAAAAGCCATTTATAATATAAAATGGAAGGA
9311                ATATTACTATATTTTACTATAAACATTATTAAAAGCCATTTATAATATAAAATGGAAGGA
                    ************************************************************

Os1892S01G025560    GTAATTAATATGGATCTCCCCCGACATGAGAATATTTTCCGATGGTGTGACGACGCCATG
9311                GTAATTAATATGGATCTCCCCCGACATGAGAATATTTTCCGATGGTGTGACGACGCCATG
                    ************************************************************
```

```
Os1892S01G025560        TAAGCTTCGGTGGGCCTGGACGGCCAGAGGTGCCAACAGCCACGTCCAACAACCCCTGGG
9311                    TAAGCTTCGGTGGGCCTGGACGGCCAGAGGTGCCAACAGCCACGTCCAACAACCCCTGGG
                        ************************************************************

Os1892S01G025560        TCCCCCCCTAACACTCCAAACAGTAGTGAGTAGTGTCTCGTCGCGTTTTAGTATTTGATG
9311                    TCCCCCCCTAACACTCCAAACAGTAGTGAGTAGTGTCTCGTCGCGTTTTAGTATTTGATG
                        ************************************************************

Os1892S01G025560        ACAAACAAAGTGTGAGTTGAGTTAGCCACCACCAACTTGCACACGAGCACATACATTTGT
9311                    ACAAACAAAGTGTGAGTTGAGTTAGCCACCACCAACTTGCACACGAGCACATACATTTGT
                        ************************************************************

Os1892S01G025560        GTCCATTCTCGCCAGTCATTTCCATCTCTACTCCTAACTCCTATCTAACGATGTAAGCGG
9311                    GTCCATTCTCGCCAGTCATTTCCATCTCTACTCCTAACTCCTATCTAACGATGTAAGCGG
                        ************************************************************

Os1892S01G025560        ATAATTTCATCATCCGTATATAAACCTGTTTGTTATAGTTAATTTCCTATATAATACTAT
9311                    ATAATTTCATCATCCGTATATAAACCTGTTTGTTATAGTTAATTTCCTATATAATACTAT
                        ************************************************************

Os1892S01G025560        AACAGTATACATTTTAAAAGAAAACAAAATTAGGATAAACAGGCCCTGCTCCTATCCATC
9311                    AACAGTATACATTTTAAAAGAAAACAAAATTAGGATAAACAGGCCCTGCTCCTATCCATC
                        ************************************************************

Os1892S01G025560        CATGGCACTTGGAAGGACCAGACTCGGTCATGCCATGCCAAGCCAAGATATGGATTATGG
9311                    CATGGCACTTGGAAGGACCAGACTCGGTCATGCCATGCCAAGCCAAGATATGGATTATGG
                        ************************************************************

Os1892S01G025560        AAGAGTAGAGAAGAGGAGAGATGAGAGATAAGCATGCGTTCTCCTCCTCGTTGGATGTGT
9311                    AAGAGTAGAGAAGAGGAGAGATGAGAGATAAGCATGCGTTCTCCTCCTCGTTGGATGTGT
                        ************************************************************

Os1892S01G025560        ATTTTGGAGGGATTTGTGTAGTAGTAGCAGCGGCGCCGCGGGGACGGATGCGGATGGTGG
9311                    ATTTTGGAGGGATTTGTGTAGTAGTAGCAGCGGCGCCGCGGGGACGGATGCGGATGGTGG
                        ************************************************************

Os1892S01G025560        CGCTTTCGGTGGCGTTTTCCTGGGGGGGTTTTGGTTTGGCGCTTGGGGGGGATGGCATGG
9311                    CGCTTTCGGTGGCGTTTTCCTGGGGGGGTTTTGGTTTGGCGCTTGGGGGGGATGGCATGG
                        ************************************************************

Os1892S01G025560        CGCGGCGTGCGGCTGCACGCCACACACGCGCGCGCACGCACGTACGTCGTCGTCGCCGCG
9311                    CGCGGCGTGCGGCTGCACGCCACACACGCGCGCGCACGCACGTACGTCGTCGTCGCCGCG
                        ************************************************************

Os1892S01G025560        GGCGGACGGTAGCTTAGGGTGGTGTGTTCCGCGCGCGGGCGCGGATTGTTCCATGCCGAT
9311                    GGCGGACGGTAGCTTAGGGTGGTGTGTTCCGCGCGCGGGCGCGGATTGTTCCATGCCGAT
                        ************************************************************

Os1892S01G025560        CGATTTGGCGCCACCCTCGCCGCGGCTCTTGTCGCGTCGTGCGCCTCTCTCGCGCGGTTT
9311                    CGATTTGGCGCCACCCTCGCCGCGGCTCTTGTCGCGTCGTGCGCCTCTCTCGCGCGGTTT
                        ************************************************************

Os1892S01G025560        GTCCTTGTCGCGTTGCTCAGCCGGCGACGGGGGCACGGACATTGGCGATGTAGCCCTGCA
9311                    GTCCTTGTCGCGTTGCTCAGCCGGCGACGGGGGCACGGACATTGGCGATGTAGCCCTGCA
                        ************************************************************

Os1892S01G025560        CGTGTCGGCCTCTCCGTTGATGAATGATGATGTATGTATGTATTTTTTTTTTGTCTGAAG
9311                    CGTGTCGGCCTCTCCGTTGATGAATGATGATGTATGTATGTATTTTTTTTTG-TCTGAAG
                        ***************************************************  *******

Os1892S01G025560        GAATTTGTGGGGAATTGTTGTGTGTGCAGGCGCTGTCGAACGGGAGGTATAAGAGCTGCC
9311                    GAATTTGTGGGGAATTGTTGTGTGTGCAGGCGCTGTCGAACGGGAGGTATAAGAGCTGCC
                        ************************************************************

Os1892S01G025560        TGCACAGGGCGGTGGTGAACCAGCGGCGGGAGCGGCGGTCGCTGGCGTTCTTCCTGTGCC
9311                    TGCACAGGGCGGTGGTGAACCAGCGGCGGGAGCGGCGGTCGCTGGCGTTCTTCCTGTGCC
                        ************************************************************

Os1892S01G025560        CGCGGGAGGACAGGGTGGTGCGGCCGCCGCCGAGCGCCGCCACGCCGCGGCACTAGCCGG
9311                    CGCGGGAGGACAGGGTGGTGCGGCCGCCGCCGAGCGCCGCCACGCCGCGGCACTAGCCGG
                        ************************************************************

Os1892S01G025560        ACTTCACCTGGGCCGACCTCATGCGCTTCACGCAGCGCCACTACCGCGCCGACACCCGCA
```

```
9311                ACTTCACCTGGGCCGACCTCATGCGCTTCACGCAGCGCCACTACCGCGCCGACACCCGCA
                    ************************************************************

Os1892S01G025560    CGCTCGACGCCTTCACGCGCTGGCTCGCGCCGCCGGCCGCCGACGCCGCCGCGACGGCGC
9311                CGCTCGACGCCTTCACGCGCTGGCTCGCGCCGCCGGCCGCCGACGCCGCCGCGACGGCGC
                    ************************************************************

Os1892S01G025560    AGGTCGAGGCGGCCAGCTGATCGCCGAACGGAACGAAACGGAACGAACAGAAGCCGATTT
9311                AGGTCGAGGCGGCCAGCTGATCGCCGAACGGAACGAAACGGAACGAACAGAAGCCGATTT
                    ************************************************************

Os1892S01G025560    TTGGCGGGGCCCACGTGGGGGATTTGCCCACGTGAGGCCCCACGTGGACAGTGGGCCCGG
9311                TTGGCGGGGCCCACGTGGGGGATTTGCCCACGTGAGGCCCCACGTGGACAGTGGGCCCGG
                    ************************************************************

Os1892S01G025560    GCGGAGGTGGCACCCACGTGGACCGCGGGCCCCGCGCCGCCTTCCAATTTTGGACCCTAC
9311                GCGGAGGTGGCACCCACGTGGACCGCGGGCCCCGCGCCGCCTTCCAATTTTGGACCCTAC
                    ************************************************************

Os1892S01G025560    CGCTGTACATATTCATATATTGCAAGAAGAAGCAAAACGTACGTGTGGGTTGGGTTGGGC
9311                CGCTGTACATATTCATATATTGCAAGAAGAAGCAAAACGTACGTGTGGGTTGGGTTGGGC
                    ************************************************************

Os1892S01G025560    TTCTCTCTATTACTAAAAAAAATATAATGGAACGACGGATGAATGGATGCTTATTTATTT
9311                TTCTCTCTATTACTAAAAAAAATATAATGGAACGACGGATGAATGGATGCTTATTTATTT
                    ************************************************************

Os1892S01G025560    ATCTAAATTGAATTCGAATTCGGCTCATGGATTTCGCGAATGTGGATGGTGGATGCCCGC
9311                ATCTAAATTGAATTCGAATTCGGCTCATGGATTTCGCGAATGTGGATGGTGGATGCCCGC
                    ************************************************************

Os1892S01G025560    CTCGATGAATCCGCTTTGTCCGATAGAGAAATTTGAATTTAAATCCGGGACCTGGATTTT
9311                CTCGATGAATCCGCTTTGTCCGATAGAGAAATTTGAATTTAAATCCGGGACCTGGATTTT
                    ************************************************************

Os1892S01G025560    GCAATGTGGACGGGTGTGCTTTGCGAAATCTGCTTTGTTCGATAGCGCTGCACAAAACAT
9311                GCAATGTGGACGGGTGTGCTTTGCGAAATCTGCTTTGTTCGATAGCGCTGCACAAAACAT
                    ************************************************************

Os1892S01G025560    GCGGTGGGCCCT------------------------------------------------
9311                GCGGTGGGCCCTGCATGAGAATCCGCTTCTTCTTTGTTGCCTTGGTAGGCGAAATCGTAT
                    ************

Os1892S01G025560    ------------------------------------------------------------
9311                ATGGTCCCAACGATTTTCTTTGTTTGGTTTCAACATAAATGGGAGTTTTTATGAATTTAG

Os1892S01G025560    ------------------------------------------------------------
9311                GCTTATCTACATCAGAGCTACTCCTAATTTGTGATATGATGAACCAATCGTGTTCTTCTC

Os1892S01G025560    ------------------------------------------------------------
9311                ATACTTGTTTAAGTTGGCCAATATAGGATTAATGCAGAGTATCCAAGGGTTTTAAGATGG

Os1892S01G025560    ------------------------------------------------------------
9311                ATCTAGTTAAGATTTGGAGAACATAATCTACAATCATCAGCAACACTAATTATAACTAAA

Os1892S01G025560    ------------------------------------------------------------
9311                TCAACTTGCCTTTTGAGTTCTCCGCAAATATCAGAACGCCTTTTTCTTTTCTTTTCTTTT

Os1892S01G025560    ------------------------------------------------------------
9311                CTTTTTTTTGAGGAGGGGGGAGCACAAAATCGGAGTGAAATTCGGGATTCCCTTCAACCA

Os1892S01G025560    ------------------------------------------------------------
9311                CTTCCAACCATGCCAAATCCCGGATGGTTTTTGTTTCTTGGCACTAAGTGATGGGTCACG

Os1892S01G025560    ------------------------------------------------------------
9311                TTTTCACAGTAGTTGATAACTTGCAACTTTCAATCACTCTATCTTCAGCTGCTCCACTGG
```

```
Os1892S01G025560        ------------------------------------------------------------
9311                    ATTCAACGTCCGTAGGAGCAGTAACTTGTCACAATGCTGAGCAGAAAATAACCGCTAGGA

Os1892S01G025560        ------------------------------------------------------------
9311                    ATATCAAATTGCACAAAATTATAATGTCACTGTTGAGTGATGAGCAGTAACTCATGAAAG

Os1892S01G025560        ------------------------------------------------------------
9311                    AATCCAAAGTTCCCATGAGTTCCAGAATGTTTGACTGATATGACAGACAAACTTTTGTAA

Os1892S01G025560        ------------------------------------------------------------
9311                    GGTTCTCCTAAATAACACAAAGAAATTTCTCGTACACTACATGGCTGTATGGATTGAACA

Os1892S01G025560        ------------------------------------------------------------
9311                    TAATTTGCCTGTGACACTTGTCACACTTGTGACACTTCCAGGTTCCAATACAACCTTACA

Os1892S01G025560        --------------------------------
9311                    GCAATGGAGCAAGAGCACAGCAAGAAGAGAAT
```

---

clustalw.dnd

(Os1892S01G025560:0.04308,9311:0.04308);

Select tree menu ⌄ Exec
