# Supplementary material for: Genetic Analysis of Lodging Resistance in 1892S Based on the T2T Genome: Providing a Genetic Approach for the Improvement of Two-Line Hybrid Rice Varieties
Source: Plants (Basel). 2025 Jun 18;14(12):1873. doi: 10.3390/plants14121873 (PMC12197197; doi:10.3390/plants14121873)
Supplement: Supplementary file 1 [file plants-14-01873-s001.zip › Supplemental File S2.pdf]

CLUSTALW Result

```
WARNING: possibly wrong combination

    ---------------------------------
    Selected type :     PROTEIN
    Query sequence:     DNA
    ---------------------------------
```

[clustalw.aln][clustalw.dnd][readme]

Select tree menu Exec

```
 CLUSTAL 2.1 Multiple Sequence Alignments


Sequence type explicitly set to Protein
Sequence format is Pearson
Sequence 1: Os06g0649800_Sdt97  3042 aa
Sequence 2: Os1892S06G004570    2878 aa
Start of Pairwise alignments
Aligning...



(Partial alignment)
Sequences (1:2) Aligned. Score: 44.5448
Guide tree file created:   [clustalw.dnd]

There are 1 groups
Start of Multiple Alignment

Aligning...
Group 1: Sequences:   2      Score:34364
Alignment Score 20229

CLUSTAL-Alignment file created  [clustalw.aln]
```

---

clustalw.aln

```
CLUSTAL 2.1 multiple sequence alignment


Os06g0649800_Sdt97      -------------------------------------------------------CCTCT
Os1892S06G004570        AAATGAGAAGTAAATAAAAGTATGTATAAAACACACAACCCAACCAACCACTACTCCTCT
                                                                           *****

Os06g0649800_Sdt97      CCTCTCCTCTCACCACCAACCTCCTCGTCCTCCTCTCCTTGGATTCCACTCCTCTCGACC
Os1892S06G004570        CCTCTCCTCTCACCACCAACCTCCTCGTCCTCCTCTCCTTGGATTCCACTCCTCTCGACC
                        ************************************************************

Os06g0649800_Sdt97      ACCACCACCAGCAGCAGCAGTAGCTGTAGCTTGCTGCTGCAGCCTGCCTCTCCTCGATCT
Os1892S06G004570        ACCACCACCACCAGCAGCAGTAGCTGTAGCTTGCTGCTGCAGCCTGCCTCTCCTCGATCT
                        ********** *************************************************

Os06g0649800_Sdt97      CCGGGCTCCCCTCTTGCCTCCGCCGTGGCGCCGTGGTGTGGTTTCTTGGTGAGCCGTGCA
Os1892S06G004570        CCGGGCTCCCCTCTTGCCTCCGCCGTGGCGCCGTGGTGTGGTTTCTTGGTGAGCCGTGCA
                        ************************************************************

Os06g0649800_Sdt97      ATGCGGGGCGGAAGCTGCCGAACCCTGGGCTGGGATTAGGGTTCTTGTCTCGGAGAGTCG
Os1892S06G004570        ATGCGGGGCGGAAGCTGCCGAACCCTGGGCTGGGATTAGGGTTCTTGTCTCGGAGAGTCG
                        ************************************************************

Os06g0649800_Sdt97      GAGGCTTTGGTGTAGTTCTTGTTTGGATTTCGAGGCTGTGTGTTCTTGGGCTTCGAGGAG
Os1892S06G004570        GAGGCTTTGGTGTAGTTCTTGTTTGGATTTCGAGGCTGTGTGTTCTTGGGCTTCGAGGAG
                        ************************************************************
```

```
Os06g0649800_Sdt97      GAGCGGTTTTCTTTGGTATCGAAGATTTGATTCTTTCGGCGGCGGGAATGGCGGCTTCCA
Os1892S06G004570        GAGCGGTTTTCTTTGGTATCGAAGATTTGATTCTTTCGGCGGCGGGAATGGCGGCTTCCA
                        ************************************************************

Os06g0649800_Sdt97      TGGCCGGAGCGCCGAGGGTCCGGTCGCTGAACGTGGCGGAGACGGACGCGGACGCGAGGC
Os1892S06G004570        TGGCCGGAGCGCCGAGGGTCCGGTCGCTGAACGTGGCGGAGACGGACGCGGACGCGAGGC
                        ************************************************************

Os06g0649800_Sdt97      CGGTGCTGGTGCCGGGTGGCAACAAGGCCAGGTCGGGCCCCGCCGCCGCCCGGAAGCCCT
Os1892S06G004570        CGGTGCTGGTGCCGGGTGGCAACAAGGCCAGGTCGGGCCCCGCCGCCGCCCGGAAGCCCT
                        ************************************************************

Os06g0649800_Sdt97      CGCCGAAGCCGCTGCGGAAGGCGGACACGGCGGCGAGGACGCCGGAGAAACCCGCCGCCG
Os1892S06G004570        CGCCGAAGCCGCTGCGGAAGGCGGACACGGCGGCGAGGACGCCGGAGAAACCCGCCGCCG
                        ************************************************************

Os06g0649800_Sdt97      CCGCCGCCGCCGCCGCTCCTCCGGCCAAGGAGGAGGAGGGCGCCAAGAAGAATGCCGGCG
Os1892S06G004570        CCGCCGCCGCCGCCGCTCCTCCGGCCAAGGAGGAGGAGGGCGCCAAGAAGAATGCCGGCG
                        ************************************************************

Os06g0649800_Sdt97      GCGGCGTGGGCAAGGGCTCGTCCCCCTTGCCGTCGCCGAGGCGGGCGCAGCCGCAGCCGC
Os1892S06G004570        GCGGCGTGGGCAAGGGCTCGTCCCCCTTGCCGTCGCCGAGGCGGGCGCAGCCGCAGCCGC
                        ************************************************************

Os06g0649800_Sdt97      CGCCGGCGAGGAAGGCGGCGCACGACGCGCCCGTGCACCTCAACCTGTCGCTCAACGCGT
Os1892S06G004570        CGCCGGCGAGGAAGGCGGCGCACGACGCGCCCGTGCACCTCAACCTGTCGCTCAACGCGT
                        ************************************************************

Os06g0649800_Sdt97      CCTGCTCCTCGGATGCCTCCGTGGAGTCGCTCCGCGGCCGGGACTCCTCCGGTGGGAGGC
Os1892S06G004570        CCTGCTCCTCGGATGCCTCCGTGGAGTCGCTCCGCGGCCGGGACTCCTCCGGTGGGAGGC
                        ************************************************************

Os06g0649800_Sdt97      TGGAGAGGAGCTGGTCCAGGGTGGCGCCCGCCGTGCCGAGGCGGGGGAAGACTCCAGTGA
Os1892S06G004570        TGGAGAGGAGCTGGTCCAGGGTGGCGCCCGCCGTGCCGAGGCGGGGGAAGACTCCAGTGA
                        ************************************************************

Os06g0649800_Sdt97      AGGCGGCGGCGGCGGCGGCGGCGGCGGAGAAGGTTGCCGCCGATGCGGAGGTTGTCGCAC
Os1892S06G004570        AGGCGGCGGCGGCGGCGGCGGCGGCGGAGAAGGTTGCCGCCGATGCGGAGGTTGTCGCAC
                        ************************************************************

Os06g0649800_Sdt97      CGGCCACACCGGAGGCGGGGAAGAGGAGGTGCGCGTGGGTGACTCCGACCAGTGGTGAGT
Os1892S06G004570        CGGCCACACCGGAGGCGGGGAAGAGGAGGTGCGCGTGGGTGACTCCGACCAGTGGTGAGT
                        ************************************************************

Os06g0649800_Sdt97      TCTTGCTTCTTGGCTTCTTGATTGTATCATTATTTGATTTTAATTTGAGTAGTAGGGAAT
Os1892S06G004570        TCTTGCTTCTTGGCTTCTTGATTGTATCATTATTTGATTTTAATTTGAGTAGTAGGGAAT
                        ************************************************************

Os06g0649800_Sdt97      ATAATTTGTGCTTAGTTCATACTCCATTTATGTGTTGCAGCTTCTGTTATTTTATCATAA
Os1892S06G004570        ATAATTTGTGCTTAGTTCATACTCCATTTATGTGTTGCAGCTTCTGTTATTTTATCATAA
                        ************************************************************

Os06g0649800_Sdt97      TTTAGTATGATTTTCCACTCAGCATACTTATTGAGAATTTTGTTGTAAATATAGGCAAAC
Os1892S06G004570        TTTAGTATGATTTTCCACTCAGCATACTTATTGAGAATTTTGTTGTAAATATAGGCAAAC
                        ************************************************************

Os06g0649800_Sdt97      CCTGCTTTAAAAATTTACCAGCAGACTAATGCTGGCTTCTTGACCCAAATAAAAATGACA
Os1892S06G004570        CCTGCTTTAAAAATTTACCAGCAGACTAATGCTGGCTTCTTGACCCAAATAAAAATGACA
                        ************************************************************

Os06g0649800_Sdt97      AATTGATCATCTAGTTGTTATGTTGTTAAACTATGTTTATCCTTTTGGAGGTGGCTCCAT
Os1892S06G004570        AATTGATCATCTAGTTGTTATGTTGTTAAACTATGTTTATCCTTTTGGAGGTGGCTCCAT
                        ************************************************************

Os06g0649800_Sdt97      CATTTTGCCTACATTGGATAAAGTGAGCATTGTCTCCTTTGTCTTGCCTATAAAGCGAGC
Os1892S06G004570        CATTTTGCCTACATTGGATAAAGTGAGCATTGTCTCCTTTGTCTTGCCTATAAAGCGAGC
                        ************************************************************

Os06g0649800_Sdt97      ACGTTCTCCATTGATGCCCTTTTCATTCTTACTACCTTCAAGGGTTTGTTGATTCCGTGT
Os1892S06G004570        ACGTTCTCCATTGATGCCCTTTTCATTCTTACTACCTTCAAGGGTTTGTTGATTCCGTGT
                        ************************************************************

Os06g0649800_Sdt97      TAGGTGTTAGCATCTGGTTATCACCTTGCATTGTTACTTCCACACAGGCCTAATATGTAA
```

```
Os1892S06G004570         TAGGTGTTAGCATCTGGTTATCACCTTGCATTGTTACTTCCACACAGGCCTAATATGTAA
                         ************************************************************

Os06g0649800_Sdt97       TTAGCTCACTAGGCTCTAGTTTGATTCATTGAATGGACATTCTAATAGAAAATGATGTGT
Os1892S06G004570         TTAGCTCACTAGGCTCTAGTTTGATTCATTGAATGGACATTCTAATAGAAAATGATGTGT
                         ************************************************************

Os06g0649800_Sdt97       TTTGCTGTATAACCATCACTTGATTAGTCCCAATTCAGAGTTTTCTGTTGCACGTGCTTG
Os1892S06G004570         TTTGCTGTATAACCATCACTTGATTAGTCCCAATTCAGAGTTTTCTGTTGCACGTGCTTG
                         ************************************************************

Os06g0649800_Sdt97       GTAGTCATCCTGCCATGAAAATCTGCCAGAATTATCTTGCAATGAACTGAAACTATCAGA
Os1892S06G004570         GTAGTCATCCTGCCATGAAAATCTGCCAGAATTATCTTGCAATGAACTGAAACTATCAGA
                         ************************************************************

Os06g0649800_Sdt97       AATTGGGTAATGAAAAGGCCACTCTACCTATAGGTAAAATGATAGGCTATATATTAATGG
Os1892S06G004570         AATTGGGTAATGAAAAGGCCACTCTACCTATAGGTAAAATGATAGGCTATATATTAATGG
                         ************************************************************

Os06g0649800_Sdt97       GTAGCTTCATCTATACCCCAAGAATACTAAAGCTTCAGGTAGGATGCTTGCTTTGTTTGA
Os1892S06G004570         GTAGCTTCATCTATACCCCAAGAATACTAAAGCTTCAGGTAGGATGCTTGCTTTGTTTGA
                         ************************************************************

Os06g0649800_Sdt97       CATTGTCACATGCTATGGTTTCCCAGATCTTTTTCTTTACCTTACTGATGAAGTATTTCT
Os1892S06G004570         CATTGTCACATGCTATGGTTTCCCAGATCTTTTTCTTTACCTTACTGATGAAGTATTTCT
                         ************************************************************

Os06g0649800_Sdt97       TGCTTTCTGCATCATTTGTCATGAAGAAAGCTGGCTTTTCAATCTCTTACTGTCCATTCA
Os1892S06G004570         TGCTTTCTGCATCATTTGTCATGAAGAAAGCTGGCTTTTCAATCTCTTACTGTCCATTCA
                         ************************************************************

Os06g0649800_Sdt97       TTGTCTCTGATCAGATCCTTGCTATGTCATCTTCCATGATGAGGAGTGGGGTGTTCCAGT
Os1892S06G004570         TTGTCTCTGATCAGATCCTTGCTATGTCATCTTCCATGATGAGGAGTGGGGTGTTCCAGT
                         ************************************************************

Os06g0649800_Sdt97       TCATGATGACAGGTATGAAAAATTGTGAATTCATGTATTCTTTCATTCTTGTTATTCAAA
Os1892S06G004570         TCATGATGACAGGTATGAAAAATTGTGAATTCATGTATTCTTTCATTCTTGTTATTCAAA
                         ************************************************************

Os06g0649800_Sdt97       GAAACTGACAGTCTGAGAATGTCGAATCATGACCGATTTCACAGGAGATTGTTTGAGCTG
Os1892S06G004570         GAAACTGACAGTCTGAGAATGTCGAATCATGACCGATTTCACAGGAGATTGTTTGAGCTG
                         ************************************************************

Os06g0649800_Sdt97       CTTGTACTGTCTGGTGCATTGGCCGAGCTCACATGGCCTGAAATTCTTAAAAGGAGGCAA
Os1892S06G004570         CTTGTACTGTCTGGTGCATTGGCCGAGCTCACATGGCCTGAAATTCTTAAAAGGAGGCAA
                         ************************************************************

Os06g0649800_Sdt97       CTTTTCAGGTATGGATTCTCTAATGTTACATAGTAGAACTGAAGAAATTATGTACTTTGT
Os1892S06G004570         CTTTTCAGGTATGGATTCTCTAATGTTACATAGTAGAACTGAAGAAATTATGTACTTTGT
                         ************************************************************

Os06g0649800_Sdt97       TCCTAACATCTGTGCCAAATCTTCCAGGGAAATTTTCGTGGACTTCGATCCTGTTGCTAT
Os1892S06G004570         TCCTAACATCTGTGCCAAATCTTCCAGGGAAATTTTCGTGGACTTCGATCCTGTTGCTAT
                         ************************************************************

Os06g0649800_Sdt97       CTCTAAAATAAATGAGAAGAAGCTTGTAGCACCAGGAAGTGTTGCCAACTCCCTTCTGTC
Os1892S06G004570         CTCTAAAATAAATGAGAAGAAGCTTGTAGCACCAGGAAGTGTTGCCAACTCCCTTCTGTC
                         ************************************************************

Os06g0649800_Sdt97       AGAGCAAAAACTCCGAGCAGTTGTTGAAAATGCTCGACAGATACTTAAGGTTAGAAGGCA
Os1892S06G004570         AGAGCAAAAACTCCGAGCAGTTGTTGAAAATGCTCGACAGATACTTAAGGTTAGAAGGCA
                         ************************************************************

Os06g0649800_Sdt97       TGCTTGATCATACACTCATACCTAGTTACAATTTCAGTTATGTATGTCTTGATTGAAGTA
Os1892S06G004570         TGCTTGATCATACACTCATACCTAGTTACAATTTCAGTTATGTATGTCTTGATTGAAGTA
                         ************************************************************

Os06g0649800_Sdt97       ACAAGGTTCCCTCAAAACTCAGATTGTAGATGAATTTGGATCCTTCGATCGATACTGTTG
Os1892S06G004570         ACAAGGTTCCCTCAAAACTCAGATTGTAGATGAATTTGGATCCTTCGATCGATACTGTTG
                         ************************************************************

Os06g0649800_Sdt97       GGGCTTTTTGAACCACAAGCCAATAGTGAGCAAATTCCGATATCCAAGGCAAGTCCCTGT
Os1892S06G004570         GGGCTTTTTGAACCACAAGCCAATAGTGAGCAAATTCCGATATCCAAGGCAAGTCCCTGT
                         ************************************************************
```

```
Os06g0649800_Sdt97      CAAGAGCCCCAAGGCAGACATGATCAGCAAAGACATGGTCCGAAGGGGTTTCCGAGGCGT
Os1892S06G004570        CAAGAGCCCCAAGGCAGACATGATCAGCAAAGACATGGTCCGAAGGGGTTTCCGAGGCGT
                        ************************************************************

Os06g0649800_Sdt97      GGGCCCAACAATCATATATTCCTTCATGCAGGCAGCAGGGCTGACCAATGATCACCTTGT
Os1892S06G004570        GGGCCCAACAATCATATATTCCTTCATGCAGGCAGCAGGGCTGACCAATGATCACCTTGT
                        ************************************************************

Os06g0649800_Sdt97      CAGTTGCTTTCGGTTCAAAGAATGCAATGAAGCTCCAACTCTTTGCACGAGTGATACTAG
Os1892S06G004570        CAGTTGCTTTCGGTTCAAAGAATGCAATGAAGCTCCAACTCTTTGCACGAGTGATACTAG
                        ************************************************************

Os06g0649800_Sdt97      CAATGCAAATTCAGAGGCAGATCTAAGTGCAGATGAACTGAGAACAAAAATCTGCAGCAA
Os1892S06G004570        CAATGCAAATTCAGAGGCAGATCTAAGTGCAGATGAACTGAGAACAAAAATCTGCAGCAA
                        ************************************************************

Os06g0649800_Sdt97      GGAGATGGCTGCAAAAGCAGAGCTGTTGAGGACGATCGGTACGCTTATCATTTCATAGGA
Os1892S06G004570        GGAGATGGCTGCAAAAGCAGAGCTGTTGAGGACGATCGGTACGCTTATCATTTCATAG--
                        **********************************************************

Os06g0649800_Sdt97      CCATGTGGAACAGTGATGCGGTTACTGTACCTGTATTGCCCATGTGTAGGTTGTGTGCTA
Os1892S06G004570        ------------------------------------------------------------
                        

Os06g0649800_Sdt97      GGTTATCCTCAAAATGAGTTTTAGGGATCAAAATGCTTTTGTGTATAAATCATTTGTGTC
Os1892S06G004570        ------------------------------------------------------------
                        

Os06g0649800_Sdt97      AGTAATGCAACCCAGTTTGTACATTGCTACCGGAGAGAAGGATGATGACCATTTTACTGA
Os1892S06G004570        ------------------------------------------------------------
                        

Os06g0649800_Sdt97      TGCAGAAACTGTGAAAAATCTTCCTCAATCAGTTAAC
Os1892S06G004570        -------------------------------------
```

---

clustalw.dnd

(Os06g0649800_Sdt97:0.27728,Os1892S06G004570:0.27728);

Select tree menu [Exec]
