# Supplementary material for: Genetic Analysis of Lodging Resistance in 1892S Based on the T2T Genome: Providing a Genetic Approach for the Improvement of Two-Line Hybrid Rice Varieties
Source: Plants (Basel). 2025 Jun 18;14(12):1873. doi: 10.3390/plants14121873 (PMC12197197; doi:10.3390/plants14121873)
Supplement: Supplementary file 1 [file plants-14-01873-s001.zip › Supplemental File S3.pdf]

CLUSTALW Result

[clustalw.aln][clustalw.dnd][readme]

Select tree menu | Exec

```
 CLUSTAL 2.1 Multiple Sequence Alignments


Sequence type explicitly set to Protein
Sequence format is Pearson
Sequence 1: Os1892S05G000700       354 aa
Sequence 2: OsR498G0511177800.01   354 aa
Start of Pairwise alignments
Aligning...



Sequences (1:2) Aligned. Score: 100
Guide tree file created:   [clustalw.dnd]

There are 1 groups
Start of Multiple Alignment

Aligning...
Group 1: Sequences:   2      Score:5846
Alignment Score 2212

CLUSTAL-Alignment file created  [clustalw.aln]
```

---

clustalw.aln

```
CLUSTAL 2.1 multiple sequence alignment


Os1892S05G000700          MVVLAKPAALEQISLVRSPSVEDNFGAGLPVVDLAADGAAGEVVRACERFGFFKVVSHGV
OsR498G0511177800.01      MVVLAKPAALEQISLVRSPSVEDNFGAGLPVVDLAADGAAGEVVRACERFGFFKVVSHGV
                          ************************************************************

Os1892S05G000700          GEGVVGRLEAEAVRFFASPQAAKDAHGPASPFGYGSKRIGRNGDMGWLEYLLLAIDGASL
OsR498G0511177800.01      GEGVVGRLEAEAVRFFASPQAAKDAHGPASPFGYGSKRIGRNGDMGWLEYLLLAIDGASL
                          ************************************************************

Os1892S05G000700          SRSSPAPSSSLRDAANKYVGAMRGMARTVLEMVAEGLGVAPRGALADMVVGDGAASDQIL
OsR498G0511177800.01      SRSSPAPSSSLRDAANKYVGAMRGMARTVLEMVAEGLGVAPRGALADMVVGDGAASDQIL
                          ************************************************************

Os1892S05G000700          RLNHYPPCPPLLQNLMPNCSPTGFGEHTDPQLISILHSNSTSGLQVALHHDADAGDHQWV
OsR498G0511177800.01      RLNHYPPCPPLLQNLMPNCSPTGFGEHTDPQLISILHSNSTSGLQVALHHDADAGDHQWV
                          ************************************************************

Os1892S05G000700          TVPPDPASFLVIVGDSLQVMTNGRMRSVRHRVVANKLKSRVSMIYFGGPPLEQRIAPLRQ
OsR498G0511177800.01      TVPPDPASFLVIVGDSLQVMTNGRMRSVRHRVVANKLKSRVSMIYFGGPPLEQRIAPLRQ
                          ************************************************************

Os1892S05G000700          LLVAGVGDGEEEEQSRYEEFTWGEYKKAAYLSRLSDNRLAPFHRQPPPVANPLA
OsR498G0511177800.01      LLVAGVGDGEEEEQSRYEEFTWGEYKKAAYLSRLSDNRLAPFHRQPPPVANPLA
                          ******************************************************
```

---

clustalw.dnd

```
(Os1892S05G000700:0,OsR498G0511177800.01:0);
```

Select tree menu | Exec
