# Supplementary material for: Genetic Analysis of Lodging Resistance in 1892S Based on the T2T Genome: Providing a Genetic Approach for the Improvement of Two-Line Hybrid Rice Varieties
Source: Plants (Basel). 2025 Jun 18;14(12):1873. doi: 10.3390/plants14121873 (PMC12197197; doi:10.3390/plants14121873)
Supplement: Supplementary file 1 [file plants-14-01873-s001.zip › Supplemental File S4.pdf]

CLUSTALW Result

```
WARNING: possibly wrong combination

   --------------------------------
   Selected type :     PROTEIN
   Query sequence:     DNA
   --------------------------------
```

[clustalw.aln][clustalw.dnd][readme]

Select tree menu | Exec

```
 CLUSTAL 2.1 Multiple Sequence Alignments


Sequence type explicitly set to Protein
Sequence format is Pearson
Sequence 1: LOC_Os07g09870_promotor     392 aa
Sequence 2: Os1892S07G004930_promotor   392 aa
Start of Pairwise alignments
Aligning...



(Partial alignment)
Sequences (1:2) Aligned. Score: 40.5612
Guide tree file created:   [clustalw.dnd]

There are 1 groups
Start of Multiple Alignment

Aligning...
Group 1: Sequences:   2      Score:4771
Alignment Score 2799

CLUSTAL-Alignment file created  [clustalw.aln]
```

---

clustalw.aln

```
CLUSTAL 2.1 multiple sequence alignment


LOC_Os07g09870_promotor      TTTGTTTCGTACCAAATTCTCAACTTGATCGTTCAAACTGAGGCTTTCGT
Os1892S07G004930_promotor    ATTGTTTCGTACCAAATTCTCAACTTGATCTTTCAAACTGAGGCTTTCGT
                             :***************************** *******************

LOC_Os07g09870_promotor      TAAACCAAGAAAATTCAGAGTGTATATATCAAACCGACGCAATTGCGACT
Os1892S07G004930_promotor    TAAACCAAGAAAATTCAGAGTGTATATATCAAACCGACGCAATTGCGACT
                             **************************************************

LOC_Os07g09870_promotor      GTGGTCTTGTGACTACATCCGGCCCCAGCCCACGTTTTCCTCTCTTTTTC
Os1892S07G004930_promotor    GTGGTCTTGTGACTACATCCGGCCCCAGCCCACGTTTTCCTCTCTTTTTC
                             **************************************************

LOC_Os07g09870_promotor      AGGGGTCCCACGGTTATTTCTCCTTTTTTAAGGGTCCCATGGTTATTTTA
Os1892S07G004930_promotor    AGGGGTCCCACGGTTATTTCTCCTTTTTTAAGGGTCCCATGGTTATTTTA
                             **************************************************

LOC_Os07g09870_promotor      TTCTTTTAGGGGTCCCACAGTTATTTTTTTTCTTTTTTTAAGGGCCCTAC
Os1892S07G004930_promotor    TTCTTTTAGGGGTCCCACAGTTATTTTTTTTCTTTTTTTAAGGGCCCTAC
                             **************************************************

LOC_Os07g09870_promotor      GGTTATTTTTTATTTTTTTAAGGGTCCCACGGTTATTTTTCAGAATGAGA
Os1892S07G004930_promotor    GGTTATTTTTTATTTTTTTAAGGGTCCCACGGTTATTTTTCAGAATGAGA
                             **************************************************
```

```
LOC_Os07g09870_promotor        TAAGACCCAGCAAAAGCAGAGACACATCCCGAGTCCCCGACTCATCCGAG
Os1892S07G004930_promotor      TAAGACCCAGCAAAAGCAGAGACACATCCCGAGTCCCCGACTCATCCGAG
                               **************************************************

LOC_Os07g09870_promotor        TCTCCGGCGAGAGCCGCCGCCGCCGACGTGCCGGACTCCGGC
Os1892S07G004930_promotor      TCTCCGGCGAGAGCCGCCGCCGCCGACGTGCCGGACTCCGGC
                               ******************************************
```

---

clustalw.dnd

(LOC_Os07g09870_promotor:0.29719,Os1892S07G004930_promotor:0.29719);

Select tree menu Exec
