# Supplementary material for: Genetic Analysis of Lodging Resistance in 1892S Based on the T2T Genome: Providing a Genetic Approach for the Improvement of Two-Line Hybrid Rice Varieties
Source: Plants (Basel). 2025 Jun 18;14(12):1873. doi: 10.3390/plants14121873 (PMC12197197; doi:10.3390/plants14121873)
Supplement: Supplementary file 1 [file plants-14-01873-s001.zip › Supplemental File S5.pdf]

CLUSTALW Result

```
WARNING: possibly wrong combination

   ---------------------------------
   Selected type :     PROTEIN
   Query sequence:     DNA
   ---------------------------------
```

[clustalw.aln][clustalw.dnd][readme]

Select tree menu Exec

```
 CLUSTAL 2.1 Multiple Sequence Alignments


Sequence type explicitly set to Protein
Sequence format is Pearson
Sequence 1: Os1892S06G022880  1363 aa
Sequence 2: Os06t0665400-01   1290 aa
Start of Pairwise alignments
Aligning...



(Partial alignment)
Sequences (1:2) Aligned. Score: 35.814
Guide tree file created:   [clustalw.dnd]

There are 1 groups
Start of Multiple Alignment

Aligning...
Group 1: Sequences:   2      Score:17387
Alignment Score 10994

CLUSTAL-Alignment file created  [clustalw.aln]
```

---

clustalw.aln

```
CLUSTAL 2.1 multiple sequence alignment


Os1892S06G022880      ATGATGAACCCTCGCCGGCTGCCGCCTCTGCCGTCGTCGACGTCGTCCGCCTCGGCAGCG
Os06t0665400-01       ATGATGAACCCTCGCCGGCTGCCGCCTCTGCCGTCGTCGACGTCGTCCGCCTCGGCAGCG
                      ************************************************************

Os1892S06G022880      GACGACATGGACCCGCGCGTGTGGCGCCGGCTGCCGCAGCCGCTGGTGGACCGCGTCCTG
Os06t0665400-01       GACGACATGGACCCGCGCGTGTGGCGCCGGCTGCCGCAGCCGCTGGTGGACCGCATCCTG
                      ******************************************************.*****

Os1892S06G022880      GCGTGCCTCCCGACGCCGTCGTTCCTCCGCCTCCGCGCCGCCTGCCGCCGCTTCTACCAC
Os06t0665400-01       GCGTGCCTCCCGACGCCGTCGTTCCTCCGCCTCCGCGCCGCCTGCCGCCGCTTCTACCAC
                      ************************************************************

Os1892S06G022880      CTCCTCTTCTCCTCCCCGTTCCTCCACTCCCACCTCCTCCTCTCCCCTCACCTCCCCTTC
Os06t0665400-01       CTCCTCTTCTCCTCCCCGTTCCTCCACTCCCACCTCCTCCTCTCCCCTCACCTCCCCTTC
                      ************************************************************

Os1892S06G022880      TTCGCCTTCGTCGTCCCCGCCGCCGGCCACCTCCTCCTCCTCGACCCCACCGCCACCGCC
Os06t0665400-01       TTCGCCTTCGTCGTCCCCGCCGCCGGCCACCTCCTCCTCCTCGACCCCACCGCCACCGCC
                      ************************************************************

Os1892S06G022880      TCCTGGTCCCGCCTCCCGCTCCCGCTCCCGCCCGTCGCCGGCGGCCCCGCCGCGTTCTCG
Os06t0665400-01       TCCTGGTCCCGCCTCCCGCTCCCGCTCCCGCCCGTCGCCGGCGGCCCCGCCGCGTTCTCG
                      ************************************************************
```

```
Os1892S06G022880        CCCGCGGCCGCGTCCGCCGGCCTGCTCGCGTTCCTGTCCGACGCGTCGGGGCACAAGACG
Os06t0665400-01         CCCGCGGCCGCGTCCGCCGGCCTGCTCGCGTTCCTGTCCGACGCGTCGGGGCACAAGACG
                        ************************************************************

Os1892S06G022880        CTGCTGCTCGCCAACCCGATCACCCGCCTCCTCGCCGCGCTCCCCATCTCCCCGACCCCG
Os06t0665400-01         CTGCTGCTCGCCAACCCGATCACCCGCCTCCTCGCCGCGCTCCCCATCTCCCCGACCCCG
                        ************************************************************

Os1892S06G022880        CGCCTCTCCCCCACCGTCGGCCTCGCCGCCGGCCCGACCTCCATCATCGCCGTCGTGGCC
Os06t0665400-01         CGCCTCTCCCCCACCGTCGGCCTCGCCGCCGGCCCGACCTCCATCATCGCCGTCGTGGCC
                        ************************************************************

Os1892S06G022880        GGGGACGACCTCGTGTCCCCCTTCGCCGTCAAGAACATCTCCGCCGACACGTTCGTCGCC
Os06t0665400-01         GGGGACGACCTCGTGTCCCCCTTCGCCGTCAAGAACATCTCCGCCGACACGTTCGTCGCC
                        ************************************************************

Os1892S06G022880        GACGCCGCCTCCGTCCCGCCCTCCGGCTTCTGGGCTCCCAGCTCCCTGCTCCCTCGCCTC
Os06t0665400-01         GACGCCGCCTCCGTCCCGCCCTCCGGCTTCTGGGCTCCCAGCTCCCTGCTCCCTCGCCTC
                        ************************************************************

Os1892S06G022880        TCCTCCCTCGATCCCGGCGCCGGCATGGCCTTCGCCTCCGGCAGGTAAGCGCGCCGCGCC
Os06t0665400-01         TCCTCCCTCGATCCCCGCGCCGGCATGGCCTTCGCCTCCGGCAG----------------
                        *************** ****************************

Os1892S06G022880        ATTGATGACGCATGGGTAAATACTCTTGCACAGATTTGCTGATCTTGCCTTGGTTGTTGT
Os06t0665400-01         ------------------------------------------------------------


Os1892S06G022880        TCTTAGGTTCTACTGCATGAGCTCGTCGCCGTTTGCGGTTCTGGTGTTCGACGTGGCGGA
Os06t0665400-01         ------GTTCTACTGCATGAGCTCGTCGCCGTTTGCGGTTCTGGTGTTCGACGTGGCGGA
                              ******************************************************

Os1892S06G022880        GAACGTATGGAGCAAGGTGCAGCCGCCGATGAGGCGGTTCCTGAGGTCGCCGGCGCTGGT
Os06t0665400-01         GAACGTATGGAGCAAGGTGCAGCCGCCGATGAGGCGGTTCCTGAGGTCGCCGGCGCTGGT
                        ************************************************************

Os1892S06G022880        GGAGCTCGGCGGCGGGAGGGAGGGAGCGGCGAGGGTGGCGCTGGTGTCGGCCGTCGAGAA
Os06t0665400-01         GGAGCTCGGCGGCGGGAGGGAGGGAGCGGCGAGGGTGGCGCTGGTGTCGGCCGTCGAGAA
                        ************************************************************

Os1892S06G022880        GAGCCGTCTCAGCGTGCCCCGCAGCGTGCGCCTGTGGACGCTGCGCGGCGGCGGCGGCG-
Os06t0665400-01         GAGCCGTCTCAGCGTGCCCCGCAGCGTGCGCCTGTGGACGCTGCGCGGCGGCGGCGGCGG
                        ***********************************************************

Os1892S06G022880        --------GTGGCGCGTGGACGGAGGTGGCGCGGATGCCGCCGGAGGTGCACGCGCAGTT
Os06t0665400-01         CGGCGGCGGTGGCGCGTGGACGGAGGTGGCGCGGATGCCGCCGGAGGTGCACGCGCAGTT
                                ****************************************************

Os1892S06G022880        CGCCGCGGCGGAGGGCGGGCGCGGGTTCGAGTGCGCGGCGCACGGCGACTACGTCGTGCT
Os06t0665400-01         CGCCGCGGCGGAGGGCGGGCGCGGGTTCGAGTGCGCGGCGCACGGCGACTACGTCGTGCT
                        ************************************************************

Os1892S06G022880        CGCGCCGCGCGGGCCCGTGGCGCAGGCGCCCACGAGCGCGCTCGTGTTCGACTCCCGCCG
Os06t0665400-01         CGCGCCGCGCGGGCCCGTGGCGCAGGCGCCCACGAGCGCGCTCGTGTTCGACTCCCGCCG
                        ************************************************************

Os1892S06G022880        CGACGAGTGGCGGTGGGCGCCGCCGTGCCCGTACGTCGTCGTCGCGCACCACGGCGGCGC
Os06t0665400-01         CGACGAGTGGCGGTGGGCGCCGCCGTGCCCGTACGTCGTCGTCGCGCACCACGGCGGCGC
                        ************************************************************

Os1892S06G022880        CGGCGCGGCGGGTTTCCGGGTGTTCGCGTACGAGCCCCGGCTGGCGACGCCGGCCATTGG
Os06t0665400-01         CGGCGCGGCGGGTTTCCGGGTGTTCGCGTACGAGCCCCGGCTGGCGACGCCGGCCATTGG
                        ************************************************************

Os1892S06G022880        CCTCCTCGACGCCACGGCGCCCGTCGCCTTGCATGGCATGCATGATGGTTAG
Os06t0665400-01         CCTCCTCGACGCCACGGCGCCCGTCGCCTTGCATGGCATGCATGATGGTTAG
                        ****************************************************
```

clustalw.dnd

(Os1892S06G022880:0.32093,Os06t0665400-01:0.32093);

Select tree menu Exec
