# Supplementary material for: Genetic Analysis of Lodging Resistance in 1892S Based on the T2T Genome: Providing a Genetic Approach for the Improvement of Two-Line Hybrid Rice Varieties
Source: Plants (Basel). 2025 Jun 18;14(12):1873. doi: 10.3390/plants14121873 (PMC12197197; doi:10.3390/plants14121873)
Supplement: Supplementary file 1 [file plants-14-01873-s001.zip › Supplemental File S6.pdf]

CLUSTALW Result

```
          WARNING: possibly wrong combination

             --------------------------------
             Selected type :     PROTEIN
             Query sequence:     DNA
             --------------------------------
```

[clustalw.aln][clustalw.dnd][readme]

Select tree menu | Exec

```
 CLUSTAL 2.1 Multiple Sequence Alignments


Sequence type explicitly set to Protein
Sequence format is Pearson
Sequence 1: Os03t0706500-01   1935 aa
Sequence 2: Os1892S03G036300  2310 aa
Start of Pairwise alignments
Aligning...



(Partial alignment)
Sequences (1:2) Aligned. Score: 65.323
Guide tree file created:   [clustalw.dnd]

There are 1 groups
Start of Multiple Alignment

Aligning...
Group 1: Sequences:   2      Score:40075
Alignment Score 15218

CLUSTAL-Alignment file created  [clustalw.aln]
```

---

clustalw.aln

```
CLUSTAL 2.1 multiple sequence alignment


Os03t0706500-01      ------------------------------------------------------------
Os1892S03G036300     GCTTTTGCCCCAACAGTAGTGTCCTTTGCCTTGTATTCTCCTCCTCTCCTCTCCTCTTGG


Os03t0706500-01      ------------------------------------------------------------
Os1892S03G036300     TGATGACTGATCGATGACCACCAGCTGCTGCACATATCATCCCCTTTCACTCCCTCTCCC


Os03t0706500-01      -----------------AAGATGGCAACACCCTGATCTCTAGCTTAGCTGCAGAGGGGAG
Os1892S03G036300     TCTCTCTCTCTCTCTATAAGATGGCAACACCCTGATCTCTAGCTTAGCTGCAGAGGGGAG
                                      *******************************************

Os03t0706500-01      AGGAACCTCACATCCAAACTCCTAGCTACAACTTGTACTAGCATCCTAAGCAACCAAGCA
Os1892S03G036300     AGGAACCTCACATCCAAACTCCTAGCTACAACTTGTACTAGCATCCTAAGCAACCAAGCA
                     ************************************************************

Os03t0706500-01      CAACCAAAGCAAGCAAGCACGAACAATTCTTTCTTCCTCTCTACCTCTAGCTGCTGCCTG
Os1892S03G036300     CAACCAAAGCAAGCAAGCACGAACAATTCTTTCTTCCTCTCTACCTCTAGCTGCTGCCTG
                     ************************************************************

Os03t0706500-01      CCTCCTAATCCTCCTACCCACCACTCCACATGAGCCCATGCTGTGTGCCTGTGTCTGTGT
Os1892S03G036300     CCTCCTAATCCTCCTACCCACCACTCCACATGAGCCCATGCTGTGTGCCTGTGTCTGTGT
                     ************************************************************
```

```
Os03t0706500-01       GTGTGT----TCTACTCCTACCATGAGAGAAGAGACCAAGCATCAACCAAGCTAGCTAGC
Os1892S03G036300      GTGTGTGTGTTCTACTCCTACCATGAGAGAAGAGACCAAGCATCAACCAAGCTAGCTAGC
                      ******    **************************************************

Os03t0706500-01       TCGTCCTCTCCTCGATCTCTACTTCTCTCTCCCACACAAGCTGAGCGCCCAGGTAGGCTG
Os1892S03G036300      TCGTCCTCTCCTCGATCTCTACTTCTCTCTCCCACACAAGCTGAGCGCCCAGGTAGGCTG
                      ************************************************************

Os03t0706500-01       CCTGCTAGGTCTCGTGCATGGCCGGACACATCTGATCATAGCCCACTACGGCACTATTCC
Os1892S03G036300      CCTGCTAGGTCTCGTGCATGGCCGGACACATCTGATCATAGCCCACTACGGCACTATTCC
                      ************************************************************

Os03t0706500-01       CCCCTTCCGCCTCGCACGCTGAGAGGTGGCCGGAGAGGGAGGGAGGCCAGCGAGCAGCAG
Os1892S03G036300      CCCCTTCCGCCTCGCACGCTGAGAGGTGGCCGGAGAGGGAGGGAGGCCAGCGAGCAGCAG
                      ************************************************************

Os03t0706500-01       TAGCAGCAGCAACGCGGCTAGGAGTAAGGAGTCCCATCAGTAAAGCATGCTTCCTTTCTT
Os1892S03G036300      TAGCAGCAGCAACGCGGCTAGGAGTAAGGAGTCCCATCAGTAAAGCATGCTTCCTTTCTT
                      ************************************************************

Os03t0706500-01       CGATTCCCCAAGCCCCATGGACATACCGCTTTACCAACAGCTTCAGCTCACCCCTCCCTC
Os1892S03G036300      CGATTCCCCAAGCCCCATGGACATACCGCTTTACCAACAGCTTCAGCTCACCCCTCCCTC
                      ************************************************************

Os03t0706500-01       TCCAAAGCCCGACCACCACCACCACCACCATTCCACCTTCTTCTACTACCACCACCACCC
Os1892S03G036300      TCCAAAGCCCGACCACCACCACCACCACCATTCCACCTTCTTCTACTACCACCACCACCC
                      ************************************************************

Os03t0706500-01       ACCTCCCTCCCCTTCCTTCCCCTCCTTCCCCTCCCCCGCCGCCGCCACGATCGCCTCGCC
Os1892S03G036300      ACCTCCCTCCCCTTCCTTCCCCTCCTTCCCCTCCCCCGCCGCCGCCACGATCGCCTCGCC
                      ************************************************************

Os03t0706500-01       GTCGCCGGCCATGCACCCCTTCATGGACTTGGAGTTGGAGCCGCATGGGCAGCAGCTGGC
Os1892S03G036300      GTCGCCGGCCATGCACCCCTTCATGGACTTGGAGTTGGAGCCGCATGGGCAGCAGCTGGC
                      ************************************************************

Os03t0706500-01       GGCGGCGGAGGAGGACGGGGCAGGCGGGCAAGGCGTCGACGCCGGGGTGCCCTTCGGCGT
Os1892S03G036300      GGCGGCGGAGGAGGACGGGGCAGGCGGGCAAGGCGTCGACGCCGGGGTGCCCTTCGGCGT
                      ************************************************************

Os03t0706500-01       CGACGGAGCGGCGGCG---GCCGCGGCGGCGAGGAAGGACCGGCACAGCAAGATAAGCAC
Os1892S03G036300      CGACGGAGCGGCGGCGGCGGCCGCGGCGGCGAGGAAGGACCGGCACAGCAAGATAAGCAC
                      ****************   *****************************************

Os03t0706500-01       CGCCGGCGGGATGAGGGACCGGCGGATGCGGCTGTCCCTCGACGTCGCCCGCAAGTTCTT
Os1892S03G036300      CGCCGGCGGGATGAGGGACCGGCGGATGCGGCTGTCCCTCGACGTCGCCCGCAAGTTCTT
                      ************************************************************

Os03t0706500-01       CGCGCTCCAGGACATGCTCGGCTTCGACAAGGCCAGCAAGACGGTGCAATGGCTCCTCAA
Os1892S03G036300      CGCGCTCCAGGACATGCTCGGCTTCGACAAGGCCAGCAAGACGGTGCAATGGCTCCTCAA
                      ************************************************************

Os03t0706500-01       CATGTCCAAGGCCGCCATCCGGGAGATCATGAGCGACGACGCCTCCTCCGTCTGCGAGGA
Os1892S03G036300      CATGTCCAAGGCCGCCATCCGGGAGATCATGAGCGACGACGCCTCCTCCGTCTGCGAGGA
                      ************************************************************

Os03t0706500-01       GGACGGCTCCAGCAGCCTCTCCGTCGACGGCAAGCAGCAGCAGCACAGCAACCCGGCGGA
Os1892S03G036300      GGACGGCTCCAGCAGCCTCTCCGTCGACGGCAAGCAGCAGCAGCACAGCAACCCGGCGGA
                      ************************************************************

Os03t0706500-01       TCGGGGCGGCGGCGCCGGGGACCACAAGGGCGCCGCTCACGGCCACAGCGACGGGAAGAA
Os1892S03G036300      TCGGGGCGGCGGCGCCGGGGACCACAAGGGCGCCGCTCACGGCCACAGCGACGGGAAGAA
                      ************************************************************

Os03t0706500-01       GCCGGCCAAGCCGAGAAGGGCAGCGGCCAACCCGAAGCCACCGCGGCGGCTGGCCAATGC
Os1892S03G036300      GCCGGCCAAGCCGAGAAGGGCAGCGGCCAACCCGAAGCCACCGCGGCGGCTGGCCAATGC
                      ************************************************************

Os03t0706500-01       GCACCCCGTCCCCGACAAGGAGTCGCGCGCCAAGGCGAGGGAGCGGGCGCGGGAGCGGAC
Os1892S03G036300      GCACCCCGTCCCCGACAAGGAGTCGCGAGCCAAGGCGAGGGAGCGGGCGCGGGAGCGGAC
                      ***************************.********************************

Os03t0706500-01       CAAGGAGAAGAACCGGATGCGGTGGGTCACCCTCGCCTCGGCAATCAGCGTCGAGGCGGC
```

```
Os1892S03G036300      CAAGGAGAAGAACCGGATGCGGTGGGTCACCCTCGCCTCGGCAATCAGCGTCGAGGCGGC
                      ************************************************************

Os03t0706500-01       CACCGCGGCGGCGGCCGCGGGGGAGGACAAGTCGCCGACGAGCCCCAGCAACAACCTGAA
Os1892S03G036300      CACCGCGGCGGCGGCCGCGGGGGAGGACAAGTCGCCGACGAGCCCCAGCAACAACCTGAA
                      ************************************************************

Os03t0706500-01       CCACTCATCGTCCACCAATCTTGTGAGCACCGAATTGGAGGACGGCTCCTCGTCAACGCG
Os1892S03G036300      CCACTCATCGTCCACCAATCTTGTGAGCACCGAATTGGAGGACGGCTCCTCGTCAACGCG
                      ************************************************************

Os03t0706500-01       CCACAACGGCGTCGGCGTCAGCGGCGGCCGGATGCAAGAAATCTCGGCGGCTAGCGAGGC
Os1892S03G036300      CCACAACGGCGTCGGCGTCAGCGGCGGCCGGATGCAAGAAATCTCGGCGGCTAGCGAGGC
                      ************************************************************

Os03t0706500-01       GAGCGACGTGATCATGGCGTTCGCCAACGGCGGCGCGTACGGCGACAGCGGCAGCTACTA
Os1892S03G036300      GAGCGACGTGATCATGGCGTTCGCCAACGGCGGCGCGTACGGCGACAGCGGCAGCTACTA
                      ************************************************************

Os03t0706500-01       CCTGCAGCAGCAGCATCAGCAGGATCAGTGGGAGCTCGGCGGCGTCGTCTACGCCAATTC
Os1892S03G036300      CCTGCAGCAGCAGCATCAGCAGGATCAGTGGGAGCTCGGCGGCGTCGTCTACGCCAATTC
                      ************************************************************

Os03t0706500-01       GCGGCACTACTGCTGATGTGATCATCCATCCACACACGAACGAACGAACGAACGGTACGG
Os1892S03G036300      GCGGCACTACTGCTGATGTGATCATCCATCCACACACGAACGAACGAACGAACGGTACGG
                      ************************************************************

Os03t0706500-01       CACTAAGATCGAACTCCTGCAGCTACATAATTATCCTTTGCTTCTCAAGAGTAATAATTC
Os1892S03G036300      CACTAAGATCGAACTCCTGCAGCTACATAATTATCCTTTGCTTCTCAAGAGTAATAATTC
                      ************************************************************

Os03t0706500-01       TTGACGTGTTAATTAATCCGGGTGTGTATTAATTCCCTCTTTATTATTTTTTCTCGCGTT
Os1892S03G036300      TTGACGTGTTAATTAATCCGGGTGTGTATTAATTCCCTCTTTATTATTTTTTCTCGCGTT
                      ************************************************************

Os03t0706500-01       TATCCGGAGTTGACTGTGGTGAAGACGAACTTTGGTTTGGTCATCGCATGGTGTGCATTG
Os1892S03G036300      TATCCGGAGTTGACTGTGGTGAAGACTAACTTTGGTTTGGTCATCGCATGGTGTGCATTG
                      ************************** *********************************

Os03t0706500-01       CATATATAGCTAGCACTATCGTCTGATCGATGATTCATC---------------------
Os1892S03G036300      CATATATAGCTAGCACTATCGTCTGATCGATGATTCATCATCAATGGAGTCGTCTAGTAT
                      ***************************************

Os03t0706500-01       ------------------------------------------------------------
Os1892S03G036300      TAATCTGTGTGCATGTTCAGATGCTCTCTGTAAACTGATAAGCTGGATACCTTGATCGCG


Os03t0706500-01       ------------------------------------------------------------
Os1892S03G036300      AGCAGGTAGATATGCCTCTCCTGAATGAGTGAAAAACAGCTGGTTTATAGTTTGCAAAAG


Os03t0706500-01       ------------------------------------------------------------
Os1892S03G036300      TTGGAGACTTGTTACTGTATTAGATCAGATCTGAAGCTAGGACACTAACTTTCCAATTTC


Os03t0706500-01       ------------------------------
Os1892S03G036300      AATCATAAATAAGAGGGAGCATATGGTGGA
```

---

clustalw.dnd

(Os03t0706500-01:0.17339,Os1892S03G036300:0.17339);

Select tree menu | Exec
