# Supplementary material for: Genetic Analysis of Lodging Resistance in 1892S Based on the T2T Genome: Providing a Genetic Approach for the Improvement of Two-Line Hybrid Rice Varieties
Source: Plants (Basel). 2025 Jun 18;14(12):1873. doi: 10.3390/plants14121873 (PMC12197197; doi:10.3390/plants14121873)
Supplement: Supplementary file 1 [file plants-14-01873-s001.zip › Supplemental File S7.pdf]

Os1892S12G021330
Os1892S08G018960
Os1892S09G022470
Os1892S05G029420
Os1892S01G028830
Os1892S01G052850
Os1892S07G018020
Os1892S07G008990
Os1892S01G010650
Os1892S03G031170
Os1892S01G044690
Os1892S02G013900
Os1892S03G038460
Os1892S05G025280
Os1892S05G018400
Os1892S09G012050
Os1892S04G024360
Os1892S01G026650
Os1892S08G003890
Os1892S03G037020
Os1892S11G014100
Os1892S03G025360
Os1892S03G006410
Os1892S01G038410
Os1892S04G011450
Os1892S07G023000
Os1892S02G015260
Os1892S04G027050
Os1892S02G032470
Os1892S11G009580
Os1892S11G019780
Os1892S02G000880
Os1892S01G049720
Os1892S03G046470
Os1892S04G012530
Os1892S05G004900
Os1892S04G026750
Os1892S07G004430
Os1892S01G025760
Os1892S02G037460
Os1892S02G016710
Os1892S08G023300
Os1892S03G030120
Os1892S09G002420
Os1892S12G005520
Os1892S01G037190
Os1892S12G000280
Os1892S03G037440
Os1892S05G026190
Os1892S07G018860
Os1892S05G007120
Os1892S04G015000
Os1892S03G040110
Os1892S12G001540

Os1892S02G041650
Os1892S04G028310
Os1892S09G016880
Os1892S06G010280
Os1892S02G003290
Os1892S12G011140
Os1892S02G028070
Os1892S08G000760
Os1892S01G003180
Os1892S09G014910
Os1892S12G019150
Os1892S07G031060
Os1892S07G026430
Os1892S12G011620
Os1892S06G004480
Os1892S09G009540
Os1892S05G027360
Os1892S03G025670
Os1892S08G004210
Os1892S01G034880
Os1892S03G031870
Os1892S11G019280
Os1892S04G003400
Os1892S02G031760
Os1892S01G033990
Os1892S05G004770
Os1892S06G007010
Os1892S11G005450
Os1892S08G004960
Os1892S01G001580
Os1892S03G036410
Os1892S03G015630
Os1892S03G026770
Os1892S09G017610
Os1892S09G022880
Os1892S04G006040
Os1892S01G014730
Os1892S07G003730
Os1892S12G011020
Os1892S06G024370
Os1892S11G019860
Os1892S02G030690
Os1892S01G050270
Os1892S07G031190
Os1892S11G023040
Os1892S09G019560
Os1892S01G015300
Os1892S06G029600
Os1892S06G032460
Os1892S07G012320
Os1892S07G018030
Os1892S05G030440
Os1892S06G030580
Os1892S08G026980

Os1892S06G011870
Os1892S01G034360
Os1892S11G018870
Os1892S01G038630
Os1892S12G020510
Os1892S06G026140
Os1892S01G049150
Os1892S06G015460
Os1892S05G000670
Os1892S10G005950
Os1892S01G023690
Os1892S11G021260
Os1892S01G018490
Os1892S03G008160
Os1892S12G012430
Os1892S02G031810
Os1892S05G008450
Os1892S01G021990
Os1892S01G038120
Os1892S04G027770
Os1892S04G030370
Os1892S11G016760
Os1892S06G018680
Os1892S04G008530
Os1892S02G022490
Os1892S04G013490
Os1892S12G001570
Os1892S03G008310
Os1892S07G021050
Os1892S02G032140
Os1892S05G020460
Os1892S09G015530
Os1892S07G008470
Os1892S12G016270
Os1892S12G009970
Os1892S11G002990
Os1892S03G000340
Os1892S06G009000
Os1892S02G007910
Os1892S10G003000
Os1892S01G005630
Os1892S01G042910
Os1892S02G042280
Os1892S10G007340
Os1892S11G015850
Os1892S05G020960
Os1892S07G026940
Os1892S11G025060
Os1892S06G020430
Os1892S09G003290
Os1892S11G021840
Os1892S12G006350
Os1892S03G012380
Os1892S01G024490

Os1892S06G018630
Os1892S02G029100
Os1892S05G019310
Os1892S06G003340
Os1892S06G023590
Os1892S05G008210
Os1892S02G042120
Os1892S11G018690
Os1892S02G008580
Os1892S11G015060
Os1892S03G036850
Os1892S05G020180
Os1892S11G019230
Os1892S06G032710
Os1892S09G010210
Os1892S12G003110
Os1892S08G010940
Os1892S02G003920
Os1892S11G013010
Os1892S09G018630
Os1892S03G019590
Os1892S03G032310
Os1892S09G017650
Os1892S08G002440
Os1892S12G023580
Os1892S12G019340
Os1892S04G013630
Os1892S06G005600
Os1892S01G028750
Os1892S02G021420
Os1892S11G010690
Os1892S05G031700
Os1892S03G033480
Os1892S02G036180
Os1892S01G014350
Os1892S05G021130
Os1892S04G029750
Os1892S12G010000
Os1892S03G014360
Os1892S04G014430
Os1892S04G015470
Os1892S03G034350
Os1892S07G011350
Os1892S06G013500
Os1892S05G002360
Os1892S02G015900
Os1892S08G026200
Os1892S11G021910
Os1892S10G017010
Os1892S08G023170
Os1892S04G003690
Os1892S03G000430
Os1892S12G005170
Os1892S03G041770

Os1892S08G006020
Os1892S09G001680
Os1892S11G022150
Os1892S08G022770
Os1892S04G007340
Os1892S12G015240
Os1892S10G018910
Os1892S04G009280
Os1892S03G027130
Os1892S03G021310
Os1892S03G024130
Os1892S05G019550
Os1892S03G036140
Os1892S07G019760
Os1892S01G007050
Os1892S03G040530
Os1892S10G004220
Os1892S07G007260
Os1892S12G015780
Os1892S04G008510
Os1892S06G028410
Os1892S03G030400
Os1892S11G010780
Os1892S03G042980
Os1892S02G009230
Os1892S06G007950
Os1892S04G028590
Os1892S03G026230
Os1892S08G019740
Os1892S08G002930
Os1892S11G005370
Os1892S09G022430
Os1892S11G004140
Os1892S02G030900
Os1892S12G010190
Os1892S07G015130
Os1892S06G030050
Os1892S11G024620
Os1892S01G004860
Os1892S05G016470
Os1892S08G021300
Os1892S01G029320
Os1892S04G009760
Os1892S08G014410
Os1892S04G014750
Os1892S09G006790
Os1892S06G026450
Os1892S07G004520
Os1892S08G023370
Os1892S07G030890
Os1892S07G023760
Os1892S01G035090
Os1892S03G024530
Os1892S08G009080

Os1892S10G003890
Os1892S05G007190
Os1892S02G039200
Os1892S03G024450
Os1892S07G014660
Os1892S03G009020
Os1892S02G007470
Os1892S12G009090
Os1892S11G027140
Os1892S01G006590
Os1892S04G013400
Os1892S02G013790
Os1892S08G028100
Os1892S11G025880
Os1892S12G000790
Os1892S02G009070
Os1892S02G018460
Os1892S04G019500
Os1892S07G007880
Os1892S01G044510
Os1892S08G009460
Os1892S05G008480
Os1892S02G036090
Os1892S04G004660
Os1892S12G001780
Os1892S02G028210
Os1892S12G010120
Os1892S08G016570
Os1892S12G023290
Os1892S12G002150
Os1892S06G007100
Os1892S11G010370
Os1892S01G053790
Os1892S09G018760
Os1892S08G022700
Os1892S06G032660
Os1892S12G017780
Os1892S06G003030
Os1892S05G008570
Os1892S12G004400
Os1892S07G015930
Os1892S09G021260
Os1892S01G048810
Os1892S03G047560
Os1892S10G021380
Os1892S09G016110
Os1892S11G006700
Os1892S02G025680
Os1892S08G022260
Os1892S01G051930
Os1892S02G034100
Os1892S04G016950
Os1892S02G003650
Os1892S03G015160

Os1892S12G015480
Os1892S01G041300
Os1892S09G002570
Os1892S09G018390
Os1892S10G015480
Os1892S02G035310
Os1892S04G029160
Os1892S02G017860
Os1892S10G011920
Os1892S07G023610
Os1892S03G019410
Os1892S09G010780
Os1892S01G003010
Os1892S05G002090
Os1892S10G014980
Os1892S01G048960
Os1892S07G006330
Os1892S12G003840
Os1892S03G016590
Os1892S02G033090
Os1892S03G030580
Os1892S06G024420
Os1892S02G019260
Os1892S07G013980
Os1892S03G027410
Os1892S02G013680
Os1892S03G007880
Os1892S10G000760
Os1892S01G045150
Os1892S04G006920
Os1892S10G014190
Os1892S12G004620
Os1892S01G025370
Os1892S01G036260
Os1892S03G022180
Os1892S05G029780
Os1892S09G020780
Os1892S06G012590
Os1892S04G000680
Os1892S01G040370
Os1892S11G003870
Os1892S12G011540
Os1892S12G018100
Os1892S09G001600
Os1892S05G001020
Os1892S12G010730
Os1892S03G007790
Os1892S01G023150
Os1892S07G021620
Os1892S08G026800
Os1892S02G004190
Os1892S01G001100
Os1892S11G011940
Os1892S02G019990

Os1892S10G020950
Os1892S09G010180
Os1892S03G045230
Os1892S11G022140
Os1892S01G004520
Os1892S09G005470
Os1892S02G032720
Os1892S05G001190
Os1892S03G027780
Os1892S09G013710
Os1892S03G015250
Os1892S11G026790
Os1892S07G003150
Os1892S12G013740
Os1892S08G026280
Os1892S10G016940
Os1892S07G001630
Os1892S01G025300
Os1892S04G026120
Os1892S07G010220
Os1892S08G007730
Os1892S09G011580
Os1892S03G047370
Os1892S01G033060
Os1892S05G022760
Os1892S05G030720
Os1892S10G018120
Os1892S11G009960
Os1892S08G001900
Os1892S03G008950
Os1892S09G007910
Os1892S01G042940
Os1892S03G012660
Os1892S10G014250
Os1892S07G014090
Os1892S01G046260
Os1892S07G019290
Os1892S11G023030
Os1892S09G006330
Os1892S12G011560
Os1892S03G037230
Os1892S06G020960
Os1892S07G018610
Os1892S06G025790
Os1892S01G013130
Os1892S08G022130
Os1892S01G005510
Os1892S02G020330
Os1892S02G030660
Os1892S02G024790
Os1892S06G030340
Os1892S11G023010
Os1892S12G002430
Os1892S05G007450

Os1892S01G014850
Os1892S12G018980
Os1892S03G045460
Os1892S11G013540
Os1892S02G001640
Os1892S08G022430
Os1892S11G018850
Os1892S05G013170
Os1892S04G031580
Os1892S03G044570
Os1892S06G003190
Os1892S10G013310
Os1892S12G006140
Os1892S03G018600
Os1892S07G001580
Os1892S10G021470
Os1892S03G016310
Os1892S08G010730
Os1892S03G046430
Os1892S11G011780
Os1892S08G020590
Os1892S05G026910
Os1892S11G015280
Os1892S01G053300
Os1892S11G020060
Os1892S05G030800
Os1892S12G016220
Os1892S06G030330
Os1892S01G053430
Os1892S05G029680
Os1892S03G016230
Os1892S05G005120
Os1892S10G007290
Os1892S02G024700
Os1892S11G017030
Os1892S03G043930
Os1892S11G012370
Os1892S08G026400
Os1892S05G023250
Os1892S11G016170
Os1892S01G035150
Os1892S04G034630
Os1892S11G003560
Os1892S03G040350
Os1892S11G000210
Os1892S12G004430
Os1892S12G001720
Os1892S04G005410
Os1892S02G035980
Os1892S06G010700
Os1892S05G009670
Os1892S08G025550
Os1892S01G007060
Os1892S06G030920

Os1892S05G026360
Os1892S01G047830
Os1892S10G011100
Os1892S12G007020
Os1892S04G014790
Os1892S04G017820
Os1892S05G002700
Os1892S11G019240
Os1892S06G028360
Os1892S11G023940
Os1892S08G000190
Os1892S04G022040
Os1892S05G020440
Os1892S06G022710
Os1892S03G034050
Os1892S07G007700
Os1892S07G005060
Os1892S11G006900
Os1892S03G025320
Os1892S11G018820
Os1892S12G001460
Os1892S12G012020
Os1892S07G020170
Os1892S08G024550
Os1892S06G003580
Os1892S08G019600
Os1892S06G018440
Os1892S07G029200
Os1892S10G009000
Os1892S06G024580
Os1892S07G018550
Os1892S01G008310
Os1892S06G005900
Os1892S08G011650
Os1892S11G006600
Os1892S12G013360
Os1892S02G007820
Os1892S03G035710
Os1892S12G023720
Os1892S07G006030
Os1892S01G042930
Os1892S05G017530
Os1892S08G017740
Os1892S03G005110
Os1892S10G005790
Os1892S10G018490
Os1892S02G005560
Os1892S11G013260
Os1892S01G053220
Os1892S09G004710
Os1892S02G030360
Os1892S01G039600
Os1892S03G005520
Os1892S01G036420

Os1892S08G025090
Os1892S03G005730
Os1892S08G021730
Os1892S10G017230
Os1892S08G017320
Os1892S10G003700
Os1892S12G011240
Os1892S08G000100
Os1892S04G006540
Os1892S10G004570
Os1892S10G018330
Os1892S04G001200
Os1892S10G017830
Os1892S05G020450
Os1892S03G042060
Os1892S11G017410
Os1892S05G016130
Os1892S05G004560
Os1892S11G003820
Os1892S07G031110
Os1892S01G033540
Os1892S03G022050
Os1892S10G010010
Os1892S06G015830
Os1892S11G022900
Os1892S08G011720
Os1892S10G006980
Os1892S12G006200
Os1892S06G019170
Os1892S01G035320
Os1892S01G043840
Os1892S03G042090
Os1892S11G002940
Os1892S07G018250
Os1892S04G003970
Os1892S09G001760
Os1892S11G014180
Os1892S09G009230
Os1892S06G003490
Os1892S12G023670
Os1892S06G020640
Os1892S10G009960
Os1892S07G023640
Os1892S01G023260
Os1892S06G027650
Os1892S07G026400
Os1892S03G000190
Os1892S06G028620
Os1892S03G022900
Os1892S11G018610
Os1892S01G039910
Os1892S03G017990
Os1892S11G011310
Os1892S02G012210

Os1892S12G001960
Os1892S09G006210
Os1892S03G036920
Os1892S12G000120
Os1892S12G017850
Os1892S08G000630
Os1892S01G046500
Os1892S12G023520
Os1892S03G010580
Os1892S11G004170
Os1892S04G036600
Os1892S03G025270
Os1892S12G011080
Os1892S11G009780
Os1892S07G018440
Os1892S07G028750
Os1892S08G003870
Os1892S06G024870
Os1892S06G002260
Os1892S12G012230
Os1892S07G018980
Os1892S01G010940
Os1892S07G016620
Os1892S02G006140
Os1892S02G036110
Os1892S05G020250
Os1892S05G012280
Os1892S08G022360
Os1892S09G004940
Os1892S03G035080
Os1892S07G020950
Os1892S03G031120
Os1892S07G018200
Os1892S05G005430
Os1892S06G001770
Os1892S07G024470
Os1892S02G023130
Os1892S08G012070
Os1892S11G012770
Os1892S10G011110
Os1892S11G027310
Os1892S07G008400
Os1892S06G017820
Os1892S11G008570
Os1892S01G046070
Os1892S12G005200
Os1892S04G014260
Os1892S06G008840
Os1892S03G003440
Os1892S02G015190
Os1892S11G023130
Os1892S01G002940
Os1892S02G016530
Os1892S06G032110

Os1892S08G018500
Os1892S02G027050
Os1892S11G025610
Os1892S06G021400
Os1892S10G020470
Os1892S07G003100
Os1892S08G012600
Os1892S04G014930
Os1892S03G001020
Os1892S12G001970
Os1892S08G013360
Os1892S10G021360
Os1892S09G009430
Os1892S05G011480
Os1892S08G004500
Os1892S10G020200
Os1892S07G016800
Os1892S07G006170
Os1892S11G003250
Os1892S02G002330
Os1892S12G008490
Os1892S07G019740
Os1892S07G001040
Os1892S11G019080
Os1892S05G016940
Os1892S12G009950
Os1892S08G015470
Os1892S02G007870
Os1892S01G028600
Os1892S03G003880
Os1892S05G002580
Os1892S03G004610
Os1892S08G017700
Os1892S10G022010
Os1892S12G022770
Os1892S03G033850
Os1892S07G026600
Os1892S08G020100
Os1892S03G005500
Os1892S08G000490
Os1892S01G039340
Os1892S03G000220
Os1892S03G002970
Os1892S03G023560
Os1892S04G018380
Os1892S03G036060
Os1892S03G047200
Os1892S08G005310
Os1892S01G009350
Os1892S04G011650
Os1892S04G002370
Os1892S06G007980
Os1892S12G004530
Os1892S10G009900

Os1892S03G004740
Os1892S04G004850
Os1892S05G003980
Os1892S01G017780
Os1892S01G022190
Os1892S07G027500
Os1892S12G021040
Os1892S02G023140
Os1892S08G015170
Os1892S02G025120
Os1892S12G004670
Os1892S02G024440
Os1892S11G002520
Os1892S03G025110
Os1892S11G001530
Os1892S07G000150
Os1892S04G007950
Os1892S07G011180
Os1892S11G009860
Os1892S01G038700
Os1892S01G029480
Os1892S11G021400
Os1892S10G009130
Os1892S10G005940
Os1892S02G015130
Os1892S04G030330
Os1892S05G001240
Os1892S05G017910
Os1892S07G020440
Os1892S03G022540
Os1892S02G026250
Os1892S07G019570
Os1892S12G022900
Os1892S05G031400
Os1892S05G018440
Os1892S11G024190
Os1892S11G027210
Os1892S10G010410
Os1892S11G002790
Os1892S12G020640
Os1892S12G013190
Os1892S07G030740
Os1892S10G005920
Os1892S08G012000
Os1892S05G021460
Os1892S05G018250
Os1892S07G013040
Os1892S07G005210
Os1892S11G000830
Os1892S10G007460
Os1892S07G000100
Os1892S09G019000
Os1892S08G012790
Os1892S09G002070

Os1892S11G013610
Os1892S12G022190
Os1892S02G003620
Os1892S10G020800
Os1892S01G042540
Os1892S05G029460
Os1892S01G027960
Os1892S10G000330
Os1892S12G010570
Os1892S10G003850
Os1892S10G000850
Os1892S04G002310
Os1892S11G024100
Os1892S12G004420
Os1892S12G019000
Os1892S02G014540
Os1892S04G016340
Os1892S05G023390
Os1892S04G000460
Os1892S08G023400
Os1892S12G012810
Os1892S01G001000
Os1892S04G032700
Os1892S02G023080
Os1892S01G026940
Os1892S03G012320
Os1892S01G013930
Os1892S07G000220
Os1892S09G015310
Os1892S12G024230
Os1892S05G004210
Os1892S04G020190
Os1892S02G001330
Os1892S12G016250
Os1892S08G011350
Os1892S01G010490
Os1892S06G032580
Os1892S09G008820
Os1892S01G034870
Os1892S08G025430
Os1892S01G025190
Os1892S04G004720
Os1892S07G006710
Os1892S01G003820
Os1892S03G022880
Os1892S05G021410
Os1892S08G005410
Os1892S04G033240
Os1892S06G012690
Os1892S11G011570
Os1892S12G005280
Os1892S11G009950
Os1892S06G003810
Os1892S08G007690

Os1892S08G023860
Os1892S07G017940
Os1892S02G024070
Os1892S12G005980
Os1892S01G031090
Os1892S06G032950
Os1892S12G012400
Os1892S05G015330
Os1892S01G018890
Os1892S04G024800
Os1892S11G005900
Os1892S11G014250
Os1892S05G031960
Os1892S12G008400
Os1892S06G006130
Os1892S12G017350
Os1892S01G008620
Os1892S08G008220
Os1892S06G025510
Os1892S09G003890
Os1892S10G013110
Os1892S11G019620
Os1892S07G007210
Os1892S01G049030
Os1892S06G017300
Os1892S07G028580
Os1892S10G017030
Os1892S06G000140
Os1892S06G032480
Os1892S04G008770
Os1892S05G010470
Os1892S05G008660
Os1892S11G014840
Os1892S05G005490
Os1892S01G004990
Os1892S05G015060
Os1892S02G000840
Os1892S12G010940
Os1892S07G001320
Os1892S09G022960
Os1892S01G025510
Os1892S05G002860
Os1892S10G016320
Os1892S02G008910
Os1892S04G001120
Os1892S03G023990
Os1892S08G014730
Os1892S11G026640
Os1892S04G012020
Os1892S12G022790
Os1892S04G014380
Os1892S08G017410
Os1892S04G036370
Os1892S04G000140

Os1892S01G039300
Os1892S11G011750
Os1892S02G017070
Os1892S04G023540
Os1892S01G032390
Os1892S07G012350
Os1892S12G011910
Os1892S06G029220
Os1892S02G015340
Os1892S12G004020
Os1892S08G003810
Os1892S12G021690
Os1892S12G007370
Os1892S09G012100
Os1892S01G005570
Os1892S08G001390
Os1892S04G000350
Os1892S07G006670
Os1892S11G021280
Os1892S07G015770
Os1892S07G022500
Os1892S08G009680
Os1892S10G001900
Os1892S12G008870
Os1892S11G023300
Os1892S05G008580
Os1892S02G028990
Os1892S04G018890
Os1892S07G010700
Os1892S04G030270
Os1892S09G020470
Os1892S06G015890
Os1892S04G007870
Os1892S01G009750
Os1892S05G020490
Os1892S05G008310
Os1892S11G013320
Os1892S01G040840
Os1892S01G030870
Os1892S04G003940
Os1892S05G025120
Os1892S01G009280
Os1892S01G024640
Os1892S03G007700
Os1892S10G007510
Os1892S04G007200
Os1892S09G000470
Os1892S02G015110
Os1892S04G032600
Os1892S02G015370
Os1892S05G024040
Os1892S07G016530
Os1892S02G005400
Os1892S12G018460

Os1892S01G041280
Os1892S05G010870
Os1892S04G032470
Os1892S05G000860
Os1892S01G050170
Os1892S09G022520
Os1892S05G010430
Os1892S08G015650
Os1892S02G007570
Os1892S04G002980
Os1892S03G005390
Os1892S06G018250
Os1892S09G019220
Os1892S04G009630
Os1892S01G027430
Os1892S03G037430
Os1892S06G018990
Os1892S05G010610
Os1892S02G029390
Os1892S12G006900
Os1892S02G014340
Os1892S01G001200
Os1892S03G000530
Os1892S06G005930
Os1892S11G024690
Os1892S11G013910
Os1892S12G011280
Os1892S08G011100
Os1892S06G022300
Os1892S09G017980
Os1892S01G020020
Os1892S04G017160
Os1892S01G038370
Os1892S11G003830
Os1892S08G020550
Os1892S08G021020
Os1892S04G007610
Os1892S05G020550
Os1892S12G020950
Os1892S03G029240
Os1892S03G031350
Os1892S07G025430
Os1892S04G005620
Os1892S11G016910
Os1892S10G020660
Os1892S11G025270
Os1892S01G006340
Os1892S01G046310
Os1892S10G009030
Os1892S04G017950
Os1892S01G021110
Os1892S05G017970
Os1892S06G005370
Os1892S01G038650

Os1892S07G005140
Os1892S04G021300
Os1892S07G007020
Os1892S07G003330
Os1892S01G011490
Os1892S05G026020
Os1892S07G030400
Os1892S08G027800
Os1892S04G030020
Os1892S03G019240
Os1892S10G009300
Os1892S05G019710
Os1892S11G018660
Os1892S03G017750
Os1892S11G011420
Os1892S09G009790
Os1892S11G006100
Os1892S11G024810
Os1892S03G039810
Os1892S05G030840
Os1892S02G008020
Os1892S09G017700
Os1892S01G038190
Os1892S01G033790
Os1892S11G009300
Os1892S03G016430
Os1892S03G001610
Os1892S04G010440
Os1892S01G036100
Os1892S02G002560
Os1892S04G013620
Os1892S12G010070
Os1892S08G026350
Os1892S07G027630
Os1892S03G032220
Os1892S04G003580
Os1892S03G020620
Os1892S06G023530
Os1892S08G020770
Os1892S08G004300
Os1892S03G018230
Os1892S02G000600
Os1892S12G015940
Os1892S11G007830
Os1892S10G020930
Os1892S07G014930
Os1892S09G020370
Os1892S02G009780
Os1892S05G024770
Os1892S10G010820
Os1892S09G007890
Os1892S08G009400
Os1892S04G009260
Os1892S03G044230

Os1892S01G047930
Os1892S02G006080
Os1892S02G033480
Os1892S04G027380
Os1892S05G029690
Os1892S07G023280
Os1892S08G021480
Os1892S03G044060
Os1892S06G024660
Os1892S11G019440
Os1892S02G023770
Os1892S07G011660
Os1892S05G007850
Os1892S05G025590
Os1892S01G040980
Os1892S11G004900
Os1892S07G009530
Os1892S05G000480
Os1892S12G020240
Os1892S01G021380
Os1892S01G012000
Os1892S11G003580
Os1892S05G025830
Os1892S09G017040
Os1892S10G016030
Os1892S01G036390
Os1892S04G003230
Os1892S02G041470
Os1892S03G036660
Os1892S02G039840
Os1892S01G051240
Os1892S08G003000
Os1892S01G006330
Os1892S03G008410
Os1892S08G018700
Os1892S01G048650
Os1892S01G012320
Os1892S08G002800
Os1892S02G010390
Os1892S03G036510
Os1892S07G030480
Os1892S12G011220
Os1892S04G036380
Os1892S05G006690
Os1892S03G028710
Os1892S12G019240
Os1892S03G019820
Os1892S10G010330
Os1892S04G028770
Os1892S03G013060
Os1892S01G001810
Os1892S11G011770
Os1892S02G015620
Os1892S08G003170

Os1892S06G013840
Os1892S03G005470
Os1892S02G029670
Os1892S10G019660
Os1892S02G023050
Os1892S04G006060
Os1892S03G014280
Os1892S02G038220
Os1892S02G020170
Os1892S02G011780
Os1892S07G002040
Os1892S08G018790
Os1892S01G033310
Os1892S05G027510
Os1892S08G007460
Os1892S07G000190
Os1892S06G030170
Os1892S12G002190
Os1892S05G029330
Os1892S11G025240
Os1892S02G042620
Os1892S03G031370
Os1892S05G008760
Os1892S07G013180
Os1892S01G019870
Os1892S07G016490
Os1892S07G030750
Os1892S08G004530
Os1892S01G006020
Os1892S09G017170
Os1892S07G010110
Os1892S06G014780
Os1892S06G023510
Os1892S07G012780
Os1892S07G014990
Os1892S04G002820
Os1892S11G025480
Os1892S08G020630
Os1892S07G017420
Os1892S12G018300
Os1892S11G005610
Os1892S11G021860
Os1892S01G040560
Os1892S06G017210
Os1892S05G021490
Os1892S11G003460
Os1892S05G013810
Os1892S05G002690
Os1892S04G014280
Os1892S08G012330
Os1892S01G050440
Os1892S03G003040
Os1892S10G016840
Os1892S01G040390

Os1892S01G040380
Os1892S04G014270
Os1892S06G010090
Os1892S07G016370
Os1892S06G024720
Os1892S02G016560
Os1892S02G030020
Os1892S06G026670
Os1892S02G023020
Os1892S08G024250
Os1892S03G008980
Os1892S04G016840
Os1892S07G009060
Os1892S04G005300
Os1892S08G027900
Os1892S06G019560
Os1892S02G032980
Os1892S11G016370
Os1892S01G001390
Os1892S08G021320
Os1892S10G014520
Os1892S06G011010
Os1892S02G023710
Os1892S07G026030
Os1892S02G027710
Os1892S12G020860
Os1892S02G027520
Os1892S06G028160
Os1892S07G002320
Os1892S02G005300
Os1892S05G017060
Os1892S04G033610
Os1892S11G022800
Os1892S03G022620
Os1892S01G019770
Os1892S06G002410
Os1892S03G043790
Os1892S01G000640
Os1892S10G008440
Os1892S04G018450
Os1892S11G016450
Os1892S05G028160
Os1892S04G001020
Os1892S03G000450
Os1892S12G019620
Os1892S02G043200
Os1892S01G050210
Os1892S01G027780
Os1892S04G011860
Os1892S03G038870
Os1892S07G028860
Os1892S07G018320
Os1892S03G032780
Os1892S07G009110

Os1892S06G004930
Os1892S02G016310
Os1892S07G006450
Os1892S07G026120
Os1892S12G013710
Os1892S01G008210
Os1892S03G018570
Os1892S03G008870
Os1892S07G028820
Os1892S05G000110
Os1892S06G026120
Os1892S11G012060
Os1892S01G052040
Os1892S04G023270
Os1892S04G023920
Os1892S03G027050
Os1892S02G042420
Os1892S06G031170
Os1892S06G002600
Os1892S07G030850
Os1892S01G015210
Os1892S06G001010
Os1892S11G005150
Os1892S12G004500
Os1892S11G018300
Os1892S09G010080
Os1892S07G017140
Os1892S06G025400
Os1892S06G026130
Os1892S02G004290
Os1892S12G014880
Os1892S07G029640
Os1892S03G023430
Os1892S11G022640
Os1892S08G025480
Os1892S01G015920
Os1892S11G010190
Os1892S01G013790
Os1892S08G017550
Os1892S02G010830
Os1892S01G050060
Os1892S09G015580
Os1892S03G032200
Os1892S06G015180
Os1892S05G001920
Os1892S10G004870
Os1892S12G008590
Os1892S02G013750
Os1892S11G014770
Os1892S03G031280
Os1892S03G016980
Os1892S04G016280
Os1892S03G017830
Os1892S03G015400

Os1892S02G019980
Os1892S06G020930
Os1892S01G014980
Os1892S01G031200
Os1892S10G003730
Os1892S11G020970
Os1892S01G003920
Os1892S01G013380
Os1892S03G022860
Os1892S06G015110
Os1892S10G013920
Os1892S01G014210
Os1892S03G004160
Os1892S11G007270
Os1892S10G010740
Os1892S02G000260
Os1892S06G026660
Os1892S11G008560
Os1892S09G001850
Os1892S02G022380
Os1892S03G010100
Os1892S06G012160
Os1892S07G019820
Os1892S10G001990
Os1892S06G024310
Os1892S04G015420
Os1892S12G014990
Os1892S09G013350
Os1892S07G030360
Os1892S01G023930
Os1892S06G022460
Os1892S03G017270
Os1892S01G054200
Os1892S11G000940
Os1892S02G010850
Os1892S03G000710
Os1892S01G012590
Os1892S01G033650
Os1892S12G006850
Os1892S02G035250
Os1892S08G016510
Os1892S03G006720
Os1892S04G035190
Os1892S12G020010
Os1892S11G005740
Os1892S02G003900
Os1892S12G008220
Os1892S11G019270
Os1892S06G003500
Os1892S05G019340
Os1892S03G031900
Os1892S04G032940
Os1892S07G020250
Os1892S12G004590

Os1892S03G022500
Os1892S04G002510
Os1892S06G029900
Os1892S08G013190
Os1892S10G008910
Os1892S01G046300
Os1892S05G026820
Os1892S11G022080
Os1892S03G001600
Os1892S01G052340
Os1892S11G006300
Os1892S08G012730
Os1892S08G013390
Os1892S05G019430
Os1892S09G015790
Os1892S01G043500
Os1892S12G011930
Os1892S02G023350
Os1892S06G029000
Os1892S10G018740
Os1892S06G020590
Os1892S11G006880
Os1892S07G005990
Os1892S08G017790
Os1892S08G005300
Os1892S11G002100
Os1892S05G016300
Os1892S07G027570
Os1892S12G022130
Os1892S06G004090
Os1892S07G017390
Os1892S08G004700
Os1892S08G008650
Os1892S12G012790
Os1892S07G010870
Os1892S08G022630
Os1892S04G023480
Os1892S01G044570
Os1892S09G011570
Os1892S09G021400
Os1892S08G019250
Os1892S08G003960
Os1892S11G015150
Os1892S09G009920
Os1892S02G007320
Os1892S06G019720
Os1892S03G001640
Os1892S07G005430
Os1892S01G025600
Os1892S01G034540
Os1892S11G018040
Os1892S10G019100
Os1892S03G031840
Os1892S05G025050

Os1892S11G005030
Os1892S11G020100
Os1892S11G023800
Os1892S10G007680
Os1892S02G000160
Os1892S02G010160
Os1892S05G014800
Os1892S08G008770
Os1892S03G039310
Os1892S06G032150
Os1892S01G008010
Os1892S03G040710
Os1892S03G002570
Os1892S01G040910
Os1892S03G027390
Os1892S08G026960
Os1892S09G018470
Os1892S03G041660
Os1892S02G029050
Os1892S03G018320
Os1892S04G020050
Os1892S07G022860
Os1892S10G008010
Os1892S09G001330
Os1892S06G032760
Os1892S07G024730
Os1892S03G040120
Os1892S01G040180
Os1892S01G004770
Os1892S08G019270
Os1892S09G006150
Os1892S03G027040
Os1892S06G012610
Os1892S01G013670
Os1892S01G034830
Os1892S05G012450
Os1892S06G012210
Os1892S07G013660
Os1892S11G013220
Os1892S02G001630
Os1892S07G015370
Os1892S07G016160
Os1892S06G021340
Os1892S06G003610
Os1892S07G013830
Os1892S05G026200
Os1892S01G003540
Os1892S06G010750
Os1892S01G052300
Os1892S06G030720
Os1892S09G009910
Os1892S05G002440
Os1892S03G024940
Os1892S12G015640

Os1892S10G013850
Os1892S08G008420
Os1892S10G011460
Os1892S11G017470
Os1892S02G017160
Os1892S11G012720
Os1892S10G007630
Os1892S09G010500
Os1892S06G028730
Os1892S09G002160
Os1892S07G022410
Os1892S12G017440
Os1892S06G010010
Os1892S04G034070
Os1892S05G010110
Os1892S03G037810
Os1892S07G027300
Os1892S01G033580
Os1892S02G021630
Os1892S03G002260
Os1892S11G015300
Os1892S05G008720
Os1892S03G032940
Os1892S03G013430
Os1892S09G020570
Os1892S10G016060
Os1892S11G024410
Os1892S06G027230
Os1892S08G008050
Os1892S09G012580
Os1892S02G010690
Os1892S05G014660
Os1892S07G015230
Os1892S01G011320
Os1892S02G012890
Os1892S09G017100
Os1892S12G009100
Os1892S07G007320
Os1892S02G009640
Os1892S11G007210
Os1892S09G000020
Os1892S01G000870
Os1892S12G009580
Os1892S06G032490
Os1892S02G000280
Os1892S01G017640
Os1892S08G026730
Os1892S01G003400
Os1892S03G020070
Os1892S04G005520
Os1892S12G014140
Os1892S12G005400
Os1892S06G002160
Os1892S06G031990

Os1892S04G013720
Os1892S12G008720
Os1892S05G024440
Os1892S10G001740
Os1892S03G004820
Os1892S06G024550
Os1892S01G003340
Os1892S05G021370
Os1892S05G020130
Os1892S03G025310
Os1892S08G015580
Os1892S02G026650
Os1892S02G029010
Os1892S08G004470
Os1892S01G052180
Os1892S01G054340
Os1892S09G011260
Os1892S12G010740
Os1892S07G011050
Os1892S07G028110
Os1892S10G000610
Os1892S03G035320
Os1892S06G006590
Os1892S01G010220
Os1892S06G030020
Os1892S06G019880
Os1892S07G030020
Os1892S11G000030
Os1892S04G034280
Os1892S08G015770
Os1892S05G004430
Os1892S02G006720
Os1892S02G030840
Os1892S02G008970
Os1892S09G003840
Os1892S06G015700
Os1892S05G016890
Os1892S05G008070
Os1892S01G033520
Os1892S08G000430
Os1892S08G023270
Os1892S05G016830
Os1892S06G011980
Os1892S08G007580
Os1892S02G025150
Os1892S01G009340
Os1892S10G018090
Os1892S03G026530
Os1892S12G004200
Os1892S06G019240
Os1892S11G008380
Os1892S11G005710
Os1892S03G019560
Os1892S08G028310

Os1892S08G013750
Os1892S11G023290
Os1892S12G004410
Os1892S02G032120
Os1892S05G007550
Os1892S07G027760
Os1892S12G021080
Os1892S04G023010
Os1892S04G015010
Os1892S12G014540
Os1892S03G009790
Os1892S05G012380
Os1892S10G001940
Os1892S01G029750
Os1892S11G017600
Os1892S08G023140
Os1892S09G022550
Os1892S08G022120
Os1892S01G041450
Os1892S03G045080
Os1892S01G045430
Os1892S11G017800
Os1892S06G017020
Os1892S07G004700
Os1892S05G018530
Os1892S12G021620
Os1892S04G003240
Os1892S04G017810
Os1892S11G006990
Os1892S07G012130
Os1892S01G029870
Os1892S09G021970
Os1892S07G016030
Os1892S03G028380
Os1892S08G010150
Os1892S08G016200
Os1892S10G003070
Os1892S01G044840
Os1892S01G008050
Os1892S07G005320
Os1892S03G029310
Os1892S08G000330
Os1892S01G022120
Os1892S02G031770
Os1892S08G009100
Os1892S04G034780
Os1892S02G002890
Os1892S04G003190
Os1892S11G012970
Os1892S02G009810
Os1892S11G002340
Os1892S12G002810
Os1892S11G018520
Os1892S12G009450

Os1892S01G021070
Os1892S01G013250
Os1892S10G017630
Os1892S06G016090
Os1892S09G003050
Os1892S03G015840
Os1892S04G031670
Os1892S08G027010
Os1892S01G013950
Os1892S06G031190
Os1892S03G022790
Os1892S08G008090
Os1892S01G022580
Os1892S05G003850
Os1892S08G026650
Os1892S03G014860
Os1892S08G001200
Os1892S07G024460
Os1892S12G015730
Os1892S03G005610
Os1892S02G037020
Os1892S02G032150
Os1892S05G025720
Os1892S04G016890
Os1892S07G000830
Os1892S02G023290
Os1892S11G019430
Os1892S01G040060
Os1892S12G013270
Os1892S01G008980
Os1892S07G013960
Os1892S07G001200
Os1892S03G020160
Os1892S04G035750
Os1892S04G003350
Os1892S01G019580
Os1892S03G032890
Os1892S03G017700
Os1892S03G028400
Os1892S05G000030
Os1892S06G032670
Os1892S03G037280
Os1892S02G008780
Os1892S06G009750
Os1892S02G021540
Os1892S07G022960
Os1892S06G007230
Os1892S06G012030
Os1892S12G005140
Os1892S02G020120
Os1892S01G021190
Os1892S07G016650
Os1892S06G018260
Os1892S06G024630

Os1892S12G000390
Os1892S12G001770
Os1892S12G008090
Os1892S08G006090
Os1892S01G029550
Os1892S02G030270
Os1892S01G030210
Os1892S06G019860
Os1892S05G012580
Os1892S11G008900
Os1892S09G022860
Os1892S04G011900
Os1892S07G015780
Os1892S05G010290
Os1892S02G012560
Os1892S10G014300
Os1892S11G003880
Os1892S02G015500
Os1892S03G045320
Os1892S04G007760
Os1892S05G000820
Os1892S05G006640
Os1892S06G020690
Os1892S08G009030
Os1892S09G009100
Os1892S01G032230
Os1892S01G019240
Os1892S06G013430
Os1892S02G031130
Os1892S07G029670
Os1892S07G008880
Os1892S12G015130
Os1892S02G014070
Os1892S03G047510
Os1892S09G020900
Os1892S09G002050
Os1892S10G005520
Os1892S01G045850
Os1892S11G021520
Os1892S01G005800
Os1892S09G018370
Os1892S03G008430
Os1892S06G023600
Os1892S07G006540
Os1892S04G009750
Os1892S11G003080
Os1892S05G022590
Os1892S02G009160
Os1892S01G031110
Os1892S09G003080
Os1892S02G005570
Os1892S03G010220
Os1892S04G033520
Os1892S05G004270

Os1892S07G030420
Os1892S12G004900
Os1892S06G000520
Os1892S06G017230
Os1892S04G011050
Os1892S07G009040
Os1892S08G002100
Os1892S06G008020
Os1892S01G000330
Os1892S12G010820
Os1892S05G000250
Os1892S06G003090
Os1892S11G010420
Os1892S07G018410
Os1892S02G020200
Os1892S09G012860
Os1892S11G015540
Os1892S01G030300
Os1892S03G032570
Os1892S03G017610
Os1892S11G018200
Os1892S03G020490
Os1892S02G026260
Os1892S06G027390
Os1892S09G015660
Os1892S02G026780
Os1892S09G009320
Os1892S11G023490
Os1892S04G011140
Os1892S02G016690
Os1892S01G016800
Os1892S02G011910
Os1892S03G005000
Os1892S01G054250
Os1892S06G021110
Os1892S12G020130
Os1892S09G016680
Os1892S02G002790
Os1892S12G010500
Os1892S11G022740
Os1892S01G028540
Os1892S05G010280
Os1892S11G004160
Os1892S06G007240
Os1892S02G002540
Os1892S02G001370
Os1892S03G013890
Os1892S08G006440
Os1892S01G037630
Os1892S12G023800
Os1892S11G007370
Os1892S02G026910
Os1892S01G033810
Os1892S06G019800

Os1892S12G019570
Os1892S10G016220
Os1892S01G003110
Os1892S08G016260
Os1892S06G024320
Os1892S07G027610
Os1892S04G015890
Os1892S09G013940
Os1892S08G010530
Os1892S06G004030
Os1892S08G025520
Os1892S11G012740
Os1892S02G039270
Os1892S03G006740
Os1892S09G001190
Os1892S02G027140
Os1892S08G001760
Os1892S08G019200
Os1892S09G000130
Os1892S06G019570
Os1892S12G000130
Os1892S01G023450
Os1892S01G003660
Os1892S10G003410
Os1892S02G027980
Os1892S01G049860
Os1892S01G030660
Os1892S11G010080
Os1892S08G005580
Os1892S04G000120
Os1892S06G027260
Os1892S03G013670
Os1892S04G010250
Os1892S05G008290
Os1892S03G037250
Os1892S06G024190
Os1892S06G008350
Os1892S06G020860
Os1892S12G020800
Os1892S06G011450
Os1892S08G014620
Os1892S02G000050
Os1892S01G017280
Os1892S04G019780
Os1892S04G010350
Os1892S04G020960
Os1892S11G009680
Os1892S02G018150
Os1892S11G012070
Os1892S06G032360
Os1892S10G014880
Os1892S11G011590
Os1892S03G046390
Os1892S01G050390

Os1892S01G027700
Os1892S08G023680
Os1892S05G001130
Os1892S10G007130
Os1892S05G021170
Os1892S08G027750
Os1892S10G009090
Os1892S10G011610
Os1892S04G029210
Os1892S06G014710
Os1892S12G024140
Os1892S11G004710
Os1892S04G024090
Os1892S06G032570
Os1892S05G005870
Os1892S05G031510
Os1892S01G051910
Os1892S03G045780
Os1892S10G011230
Os1892S05G030500
Os1892S05G016140
Os1892S10G012540
Os1892S10G008960
Os1892S11G021380
Os1892S05G014690
Os1892S01G029240
Os1892S01G019060
Os1892S01G038230
Os1892S01G028070
Os1892S05G030670
Os1892S07G019930
Os1892S12G013170
Os1892S10G022070
Os1892S02G027670
Os1892S11G026210
Os1892S02G014380
Os1892S03G045500
Os1892S05G011640
Os1892S12G010290
Os1892S12G003950
Os1892S05G029950
Os1892S12G016240
Os1892S03G008050
Os1892S08G012830
Os1892S10G010840
Os1892S10G010020
Os1892S02G029500
Os1892S02G013100
Os1892S11G002800
Os1892S01G020720
Os1892S06G027460
Os1892S07G014400
Os1892S01G015750
Os1892S08G025160

Os1892S11G009240
Os1892S01G036720
Os1892S07G012310
Os1892S03G013600
Os1892S10G020740
Os1892S09G009380
Os1892S10G016550
Os1892S03G018790
Os1892S07G020730
Os1892S07G008760
Os1892S03G047880
Os1892S01G037270
Os1892S05G009120
Os1892S03G034540
Os1892S01G047130
Os1892S04G001560
Os1892S02G031610
Os1892S06G016750
Os1892S12G012450
Os1892S09G013720
Os1892S04G011640
Os1892S01G026920
Os1892S02G024130
Os1892S03G014250
Os1892S06G026630
Os1892S04G031590
Os1892S06G004580
Os1892S09G022130
Os1892S12G017680
Os1892S02G030970
Os1892S07G011040
Os1892S03G028170
Os1892S09G013870
Os1892S03G009250
Os1892S05G000050
Os1892S12G010060
Os1892S01G018520
Os1892S08G003910
Os1892S02G035230
Os1892S08G002580
Os1892S12G013760
Os1892S11G019640
Os1892S11G000570
Os1892S06G006290
Os1892S01G031820
Os1892S07G022680
Os1892S06G004890
Os1892S04G033260
Os1892S08G015510
Os1892S01G020730
Os1892S05G004800
Os1892S11G014540
Os1892S01G032670
Os1892S04G027470

Os1892S04G001720
Os1892S10G021840
Os1892S08G002210
Os1892S11G005110
Os1892S11G002120
Os1892S01G015890
Os1892S06G009550
Os1892S11G001550
Os1892S12G008650
Os1892S11G022830
Os1892S06G012650
Os1892S12G007350
Os1892S02G040650
Os1892S08G010430
Os1892S10G002390
Os1892S01G037490
Os1892S04G027070
Os1892S04G012400
Os1892S03G033080
Os1892S12G017090
Os1892S08G023780
Os1892S07G016260
Os1892S01G007080
Os1892S05G005550
Os1892S12G005340
Os1892S03G002720
Os1892S12G021880
Os1892S06G002730
Os1892S08G010670
Os1892S11G016410
Os1892S11G022440
Os1892S02G036640
Os1892S03G000480
Os1892S03G038490
Os1892S11G023500
Os1892S02G018550
Os1892S11G015200
Os1892S01G012220
Os1892S10G009160
Os1892S11G025720
Os1892S04G029960
Os1892S05G012770
Os1892S07G011490
Os1892S03G011430
Os1892S06G027130
Os1892S03G047160
Os1892S01G012630
Os1892S01G050400
Os1892S03G012020
Os1892S04G032690
Os1892S07G017890
Os1892S09G015150
Os1892S03G042890
Os1892S06G007550

Os1892S05G001510
Os1892S12G020310
Os1892S03G038440
Os1892S02G037590
Os1892S12G018330
Os1892S10G021190
Os1892S12G009510
Os1892S10G004730
Os1892S11G005520
Os1892S11G019110
Os1892S07G029550
Os1892S10G006100
Os1892S12G019020
Os1892S08G009240
Os1892S01G027300
Os1892S11G004980
Os1892S11G018160
Os1892S01G035400
Os1892S10G014500
Os1892S07G013170
Os1892S07G011860
Os1892S06G002280
Os1892S03G031020
Os1892S05G000630
Os1892S07G010820
Os1892S06G017090
Os1892S11G008950
Os1892S04G035640
Os1892S06G027870
Os1892S11G022760
Os1892S01G000500
Os1892S08G020680
Os1892S01G044050
Os1892S06G015760
Os1892S07G020020
Os1892S01G015350
Os1892S01G013530
Os1892S10G020270
Os1892S11G026020
Os1892S08G016220
Os1892S03G038150
Os1892S05G000550
Os1892S08G017990
Os1892S01G015840
Os1892S02G009150
Os1892S08G010030
Os1892S01G041180
Os1892S03G005080
Os1892S09G012130
Os1892S04G015120
Os1892S01G025870
Os1892S09G010220
Os1892S12G011040
Os1892S04G035940

Os1892S01G049820
Os1892S11G013290
Os1892S04G008010
Os1892S11G027420
Os1892S11G006560
Os1892S10G009340
Os1892S12G002110
Os1892S08G000090
Os1892S01G032950
Os1892S03G026190
Os1892S02G040810
Os1892S03G024700
Os1892S04G024690
Os1892S05G026660
Os1892S08G003880
Os1892S08G008790
Os1892S06G001300
Os1892S04G029000
Os1892S01G021180
Os1892S11G006720
Os1892S09G019650
Os1892S03G018720
Os1892S11G002070
Os1892S03G016480
Os1892S03G044160
Os1892S10G010940
Os1892S09G020040
Os1892S01G034740
Os1892S07G030230
Os1892S01G026420
Os1892S11G026950
Os1892S05G019480
Os1892S02G040140
Os1892S05G032280
Os1892S02G032230
Os1892S12G000780
Os1892S06G021950
Os1892S11G016510
Os1892S04G009540
Os1892S02G001720
Os1892S06G017760
Os1892S03G038570
Os1892S02G035790
Os1892S01G002350
Os1892S07G018350
Os1892S11G004940
Os1892S12G018760
Os1892S07G008910
Os1892S01G000160
Os1892S11G012120
Os1892S03G021280
Os1892S01G050690
Os1892S08G008250
Os1892S10G017680

Os1892S06G020520
Os1892S02G012160
Os1892S01G034770
Os1892S06G017200
Os1892S05G011000
Os1892S02G023560
Os1892S01G019610
Os1892S07G016390
Os1892S04G012250
Os1892S10G001280
Os1892S01G034280
Os1892S07G026860
Os1892S08G018920
Os1892S11G018900
Os1892S07G015960
Os1892S06G001340
Os1892S02G011600
Os1892S03G011920
Os1892S01G017120
Os1892S01G004760
Os1892S03G046690
Os1892S05G010660
Os1892S05G031340
Os1892S11G006830
Os1892S10G012130
Os1892S04G015770
Os1892S10G018040
Os1892S02G012330
Os1892S11G003720
Os1892S01G001730
Os1892S07G008940
Os1892S09G020920
Os1892S07G027450
Os1892S01G031960
Os1892S01G021930
Os1892S04G018010
Os1892S12G021960
Os1892S12G008310
Os1892S12G020790
Os1892S07G018880
Os1892S11G024570
Os1892S04G010820
Os1892S06G016840
Os1892S11G021450
Os1892S03G018440
Os1892S01G033180
Os1892S11G005790
Os1892S08G014680
Os1892S04G028150
Os1892S03G020040
Os1892S01G052840
Os1892S02G011690
Os1892S04G031690
Os1892S06G025060

Os1892S12G015430
Os1892S02G014660
Os1892S02G013300
Os1892S05G002030
Os1892S07G002600
Os1892S02G007810
Os1892S12G011980
Os1892S11G023730
Os1892S11G020280
Os1892S03G046800
Os1892S04G006740
Os1892S06G026370
Os1892S02G016610
Os1892S11G002840
Os1892S03G010950
Os1892S10G006570
Os1892S08G007260
Os1892S04G028870
Os1892S12G006920
Os1892S05G000510
Os1892S08G018420
Os1892S05G029080
Os1892S11G014920
Os1892S02G007940
Os1892S11G006550
Os1892S01G026950
Os1892S08G021760
Os1892S06G004160
Os1892S11G010070
Os1892S08G003390
Os1892S11G026360
Os1892S08G027050
Os1892S08G003730
Os1892S02G018660
Os1892S01G022280
Os1892S04G006530
Os1892S01G008730
Os1892S02G018430
Os1892S02G023490
Os1892S02G031390
Os1892S04G010850
Os1892S06G021230
Os1892S07G030320
Os1892S08G000390
Os1892S06G027600
Os1892S03G004270
Os1892S08G020370
Os1892S01G054580
Os1892S02G021470
Os1892S03G029200
Os1892S01G030020
Os1892S05G025240
Os1892S11G013230
Os1892S12G013060

Os1892S02G020490
Os1892S01G012520
Os1892S04G025830
Os1892S08G008620
Os1892S06G007780
Os1892S05G010540
Os1892S04G001830
Os1892S01G010580
Os1892S07G017840
Os1892S11G014360
Os1892S01G032940
Os1892S07G015090
Os1892S04G000300
Os1892S07G030500
Os1892S09G008810
Os1892S08G024020
Os1892S04G035350
Os1892S10G019790
Os1892S05G013460
Os1892S04G032950
Os1892S12G009900
Os1892S01G038640
Os1892S04G021050
Os1892S07G011460
Os1892S06G032860
Os1892S01G038530
Os1892S09G004290
Os1892S06G000250
Os1892S10G018010
Os1892S02G022290
Os1892S04G003380
Os1892S06G030530
Os1892S06G026740
Os1892S04G024240
Os1892S10G002670
Os1892S12G014490
Os1892S07G021150
Os1892S07G000310
Os1892S11G022020
Os1892S11G001180
Os1892S12G004870
Os1892S02G011180
Os1892S04G005220
Os1892S03G029390
Os1892S02G032780
Os1892S11G014960
Os1892S12G019370
Os1892S05G016550
Os1892S09G003320
Os1892S01G008000
Os1892S03G004810
Os1892S01G034810
Os1892S04G012130
Os1892S06G017500

Os1892S04G025630
Os1892S10G010670
Os1892S02G007840
Os1892S09G003240
Os1892S02G023780
Os1892S09G015040
Os1892S09G009800
Os1892S11G018730
Os1892S01G036910
Os1892S12G002680
Os1892S03G016970
Os1892S06G028800
Os1892S08G021690
Os1892S12G012670
Os1892S03G010530
Os1892S01G027820
Os1892S12G024040
Os1892S04G036500
Os1892S02G030230
Os1892S03G013090
Os1892S05G026870
Os1892S05G031040
Os1892S11G004300
Os1892S02G015540
Os1892S05G031910
Os1892S04G034120
Os1892S02G010050
Os1892S05G000410
Os1892S01G002520
Os1892S07G018540
Os1892S08G014940
Os1892S05G009200
Os1892S06G013790
Os1892S02G024270
Os1892S11G006160
Os1892S10G012850
Os1892S11G008580
Os1892S12G013040
Os1892S01G045760
Os1892S08G027950
Os1892S06G022720
Os1892S07G030630
Os1892S11G006840
Os1892S01G014930
Os1892S08G007940
Os1892S03G028880
Os1892S05G016010
Os1892S02G041390
Os1892S01G020850
Os1892S03G006260
Os1892S07G026180
Os1892S01G039440
Os1892S12G011640
Os1892S04G024220

Os1892S04G006990
Os1892S10G004330
Os1892S11G017590
Os1892S02G006780
Os1892S05G030870
Os1892S05G010320
Os1892S08G011480
Os1892S06G015130
Os1892S08G017730
Os1892S03G023270
Os1892S11G011840
Os1892S06G013660
Os1892S11G013850
Os1892S03G014140
Os1892S03G032910
Os1892S04G002030
Os1892S01G004340
Os1892S12G009120
Os1892S08G008460
Os1892S05G004790
Os1892S11G024710
Os1892S05G023350
Os1892S07G018080
Os1892S01G023710
Os1892S03G034090
Os1892S06G028060
Os1892S03G030270
Os1892S11G020220
Os1892S06G002980
Os1892S05G026990
Os1892S08G015980
Os1892S12G018130
Os1892S07G018730
Os1892S09G020990
Os1892S06G030350
Os1892S08G023730
Os1892S11G018800
Os1892S07G027660
Os1892S06G020190
Os1892S03G015320
Os1892S05G017430
Os1892S06G001110
Os1892S01G022300
Os1892S03G039450
Os1892S05G007560
Os1892S01G053670
Os1892S12G011790
Os1892S12G002900
Os1892S01G018000
Os1892S03G014180
Os1892S08G020530
Os1892S06G025980
Os1892S01G027620
Os1892S12G005620

Os1892S04G014470
Os1892S12G018380
Os1892S01G002020
Os1892S08G015870
Os1892S04G026480
Os1892S07G014310
Os1892S10G001070
Os1892S02G035510
Os1892S04G016700
Os1892S07G027420
Os1892S05G017010
Os1892S07G003710
Os1892S06G012990
Os1892S10G021600
Os1892S05G012500
Os1892S08G021780
Os1892S02G007050
Os1892S03G030300
Os1892S11G005930
Os1892S07G002780
Os1892S12G022850
Os1892S05G011430
Os1892S06G016480
Os1892S04G013240
Os1892S08G028320
