# Supplementary material for: Genetic Analysis of Lodging Resistance in 1892S Based on the T2T Genome: Providing a Genetic Approach for the Improvement of Two-Line Hybrid Rice Varieties
Source: Plants (Basel). 2025 Jun 18;14(12):1873. doi: 10.3390/plants14121873 (PMC12197197; doi:10.3390/plants14121873)
Supplement: Supplementary file 1 [file plants-14-01873-s001.zip › Supplemental File S8.pdf]

Pfams:
DIOX_N, 2OG-FeII_Oxy
Non-core Gene Id:
Os1892S06G003610
Os1892S01G008000

Pfams:
F-box
Non-core Gene Id:
Os1892S02G028210
Os1892S01G047130
Os1892S11G002120
Os1892S12G011020
Os1892S11G015200
Os1892S08G010030
Os1892S04G002030
Os1892S06G026660
Os1892S10G019100
Os1892S02G023290
Os1892S06G003490
Os1892S04G027770
Os1892S11G015300
Os1892S10G011110
Os1892S06G032710
Os1892S07G002040
Os1892S10G009960
Os1892S06G018250
Os1892S03G003040
Os1892S02G004190
Os1892S11G027210
Os1892S02G035510
Os1892S07G012780
Os1892S12G015430
Os1892S08G004700
Os1892S11G013220
Os1892S05G008480
Os1892S11G027310
Os1892S07G012320
Os1892S08G025550
Os1892S07G006330
Os1892S06G023530

Pfams:
Adenine_glyco
Non-core Gene Id:
Os1892S01G054340
